# A Flexible-Horizon Clinical Decision Support Model for Dementia Early Detection and Risk Prediction in Secondary Care: A Responsible AI Approach

**DOI:** 10.1007/s10916-026-02458-2

**Published:** 2026-09-15

**Authors:** Adnane Ez-zizi, Kalyan Seelam, Lisa Leggett, Bilal R. Malik

**Affiliations:** 1 School of Business, Arts, Social Sciences and Technology, University of Suffolk, Ipswich, IP4 1QJ UK; 2 Digital Futures Institute, University of Suffolk, Ipswich, IP4 1QJ UK; 3 Memory Assessment & Support Service, South West Yorkshire Partnership NHS Foundation Trust, Wakefield, WF1 3SP UK; 4 Older Adults Mental Health Services, South West Yorkshire Partnership NHS Foundation Trust, Wakefield, WF1 3SP UK; 5 Biomedical and Forensic Sciences, School of Sciences, University of Derby, Derby, DE22 1GB UK; 6 Neurodegeneration and Healthy Ageing Cluster, University of Derby, Derby, DE22 1GB UK

**Keywords:** Dementia, Machine learning, Responsible AI, Survival analysis, National Alzheimer’s Coordinating Center Uniform Data Set (NACC UDS)

## Abstract

Existing machine learning (ML) models for dementia detection typically use fixed prediction windows and often lack clinically informed variable selection and responsible AI principles, thereby limiting performance, generalisability and clinical trust. We aimed to develop a single clinically informed model capable of both diagnosing dementia and predicting its onset over flexible time horizons up to 10 years. We trained our ML model on the NACC Uniform Data Set using 20 inputs compatible with UK National Healthcare Service secondary-care workflows, including a *HORIZON* feature encoding months from baseline. We also integrated explainability and fairness analyses. Same-visit classification achieved sensitivity of 0.951 (95% confidence interval 0.947–0.954), specificity of 0.810 (0.805–0.814), a geometric mean (G-mean) of 0.877 (0.874–0.880) and an area under the receiver operating characteristic curve (AUC) of 0.959 (0.957–0.960). Across future horizons from 24 to 120 months, sensitivity/specificity ranged between 0.808/0.856 at 24 months and 0.833/0.706 at 120 months, while AUCs were 0.906 and 0.851. Compared with a Cox proportional hazards model, our flexible-horizon ML model achieved a higher G-mean at four of the five evaluated horizons and higher specificity from 48 months onwards, whereas Cox achieved greater sensitivity from 48 months onwards. Shapley additive explanations highlighted functional independence, prediction horizon and cognitive measures as influential. Exploratory subgroup analyses showed broadly similar balanced discrimination across sex, race and age groups, although sensitivity–specificity trade-offs varied. An interactive web application was also developed for demonstration and clinical feedback. These findings support the feasibility of flexible-horizon, clinically-informed and responsible ML-based decision support for dementia early detection; however, external validation, recalibration and prospective clinical evaluation are required before deployment.

**Supplementary Information:**

The online version contains supplementary material available at https://doi.org/10.1007/s10916-026-02458-2.

## Introduction

Dementia is a syndrome resulting from various diseases including Alzheimer’s, and is marked by progressive deterioration of cognitive functions such as memory, speech and judgment [1]. It ranks among the most significant contributors to escalating healthcare costs worldwide, particularly in ageing populations [2]. Nearly 60 million individuals globally have dementia, a figure expected to more than double by 2050 [2, 3]. Given that irreversible brain damage typically occurs long before a clinical diagnosis is made, the best defence against dementia is to implement effective early detection methods combined with risk-reducing lifestyle interventions.

Conventional diagnostic approaches that combine psychometric, anatomical, imaging and lifestyle data can provide highly accurate dementia diagnosis and prediction [4]. However, these methods can be time- and resource-intensive, often requiring multiple specialist referrals and resulting in delays (an issue further exacerbated by the COVID-19 backlog [5]). In response, various simplified toolkits have been proposed, such as CAIDE (Cardiovascular Risk Factors, Aging, and Incidence of Dementia) [6] and BDSI (Brief Dementia Screening Indicator) [7]. However, these toolkits have several limitations. For example, both CAIDE and BDSI were designed for specific age groups (CAIDE for middle-aged adults, BDSI for older adults) and operate over pre-specified time horizons (20 years for CAIDE and 6 years for BDSI; which also means they cannot be used for immediate diagnosis). Moreover, these tools were shown to have a poor ratio of detected dementia to false positive results, and may provide marginal to no additional discriminative value when compared to prediction based on age alone [8, 9].

A promising avenue for addressing these limitations involves leveraging machine learning (ML) and the increasingly available large-scale datasets (e.g. NACC Uniform Data Set (UDS) [10], UK Biobank [11]). Recent ML models trained on clinical data have shown potential for predicting dementia over preset time horizons [12]. For example, James and colleagues [13] developed one of the earliest ML models trained and tested on the NACC UDS with a 29-month prediction window, and using 22 variables (later reduced to five, albeit with sensitivity dropping from 0.45 to 0.28). Another NACC UDS-based study combined both unsupervised and supervised learning, and capitalised on longitudinal information, for a one-year prediction, reporting a sensitivity of 0.93 and a specificity of 0.58 [14]. Besides, in [15], a 12-year dementia prediction model is trained on the AGES-Reykjavik dataset [16] using 16 clinically accessible variables, and achieving 0.61 sensitivity and 0.69 specificity. These and other existing ML models (e.g., [17–19]) often struggle to balance high sensitivity and specificity.

We hypothesise that this challenge stems largely from rigidly defining a single prediction horizon—a practice that discards valuable data and can introduce sampling bias if right-censored data is not handled properly. For example, in most previous works, dementia onset within a fixed time horizon (e.g. 24 months) is inferred using follow-up data that extends beyond the horizon when such data is available. This practice may artificially inflate the proportion of dementia cases in both the training and test sets. More broadly, discarding data beyond a preset horizon may be limiting because the association patterns between dementia risk and some explanatory variables (e.g. family history, neuropsychological test scores) are likely to remain largely consistent irrespective of the prediction horizon, and thus, such additional data should be expected to improve ML performance.

A more principled way to analyse time-to-event outcomes is through survival analysis, for example using the Cox proportional hazards model. However, such a model solves a more demanding regression-type problem in which the aim is to estimate time to dementia onset, often relies on strong assumptions such as proportional hazard functions, non-informative censoring and independence of observations, and are supported by less robust and less widely adopted software ecosystems than standard ML approaches [20]. An alternative approach that has recently gained attention is to recast survival (regression) analysis as a classification problem through a data augmentation framework known as survival stacking [21]. In survival stacking, the data are reorganised so that each individual contributes multiple rows, one for each event time at which they are still at risk. Each row is labeled 1 if the event occurs at that time and 0 otherwise, turning the survival problem into a standard binary classification task. In [21], for example, logistic regression with survival stacking is shown to be approximately equivalent to the Cox proportional hazards model. The approach proposed in this paper is conceptually related. Specifically, we introduce a new variable, *HORIZON*, representing the prediction window relative to each recorded patient visit (equivalent to survival time), while the target outcome is whether dementia is diagnosed at a visit (equivalent to the occurrence of the survival event). Unlike conventional survival stacking, however, we do not generate synthetic samples between observed survival times, and instead, exploit the rich multi-visit structure already available in the NACC UDS.

Another limitation in the literature is the limited integration of multi-facet responsible AI approaches when developing ML models for dementia, including both explainability and fairness dimensions. While explainable AI is gaining traction in this area [22–24], dementia screening models rarely address the possible existence of model biases against underrepresented groups based on protected attributes, such as race, sex and age—a crucial consideration before deploying such tools in healthcare settings (see [25] for an exception).

To address these gaps, we propose a novel flexible-horizon ML model based on 20 routinely collected, objective and easy-to-measure variables selected for compatibility with the UK National Healthcare Service (NHS) secondary care workflows. Unlike previously reported approaches designed for a single prediction window, our model is designed to handle both point-of-assessment classification (*HORIZON* = 0) and prediction across future time horizons within the same modelling framework. Our aim is not to claim head-to-head superiority over all previously published methods, but rather to examine whether a single clinically informed model could achieve competitive performance while incorporating explainability and fairness analyses. Trained on NACC UDS data, the proposed model targets both high sensitivity and specificity and has the potential to support earlier dementia screening and diagnosis. This, in turn, may enable more timely intervention and lifestyle modification for individuals at risk. Because the model relies on simple and routinely collected variables, it has the potential to be used by junior doctors and medical assistants, thereby reducing the burden on dementia specialists and minimising the need for prolonged, in-depth assessments. To facilitate demonstration, clinical feedback and future validation work, we have also implemented the model as an interactive web application, available at https://dementiaapp.aezzizi.com.^1^

Importantly, we frame this model as a secondary care decision-support tool for early detection and short- to medium-term risk stratification among patients presenting with memory, cognitive or functional concerns. Such concerns may arise from dementia but also from other potentially temporary or non-dementia causes, including depressive episodes, medication effects, and other neurological or medical conditions. At present, the cognitive, functional and clinical inputs required by the model are more routinely available within secondary care memory assessment pathways, and this is therefore the setting in which the model currently fits most naturally. The model is not intended to serve as a pure population-level long-term dementia risk model for asymptomatic individuals. However, as care pathways evolve and more structured cognitive and functional assessments become available in primary care, the model could potentially be evaluated for use earlier in the clinical pathway.

## Methods

### Data Source

This study used the Uniform Data Set (UDS) version 3.0, obtained from the National Alzheimer’s Coordinating Center (NACC) in March 2023 [10]. The dataset was curated through collaboration with over 42 Research Centres for Alzheimer’s Disease across the United States. The current version includes data from 47,400 patients who visited these centres between June 2005 and February 2023, with an average of 3.3 visits per patient (min = 1, max = 18). Written informed consent was obtained from all participants and co-participants, and ethical approval was secured prior to data collection. In total, the dataset contains 155,997 samples and 1,024 variables, covering a wide range of themes such as demographic information, medical history, family medical history and neuropsychological tests. For the purposes of this study, we used the variable *DEMENTED* as the target for classifying patients with and without dementia in our ML models.

### Feature Selection

Feature selection was conducted in five stages to identify the most relevant and efficient variables for dementia screening.

**Stage 1**: Variables were filtered based on their alignment with standard diagnostic protocols in NHS secondary care. Specifically, our clinical co-author (consultant psychiatrist in an NHS memory assessment and support service) reviewed candidate NACC variables against two criteria: (i) their routine availability in secondary-care dementia assessment, and (ii) minimal reliance on subjective expert judgment to ensure that they could be collected by junior clinical staff or medical assistants. This process reduced the number of variables to 107 (see Supplemental Table 1 for details), categorised as follows: demographic information (5 variables), medications (4 variables), subject health history (17 variables), family health history (3 variables), blood pressure information (1 variable), Hachinski ischemic score and cerebrovascular disease (5 variables), unified Parkinson’s disease rating scale (11 variables), geriatric depression scale (17 variables), functional activities questionnaire (10 variables), physical and neurological exam findings (7 variables), neuropsychological battery scores (26 variables), and clinician diagnosis (1 variable). Although one clinician diagnosis variable was included in the initial clinically reviewed candidate set, no diagnosis derived or clinician judgement variables were included as predictors in model training. The variable DEMENTED was used only as the target outcome.

**Stage 2**: We constructed flexible-horizon samples so that each row answered the question: given baseline information only, would this patient have dementia by a given time horizon? To achieve this, a new variable, *HORIZON*, was created to represent the number of months between a patient’s first visit and each subsequent visit. The introduction of this variable is key to enabling our ML models to diagnose and/or predict dementia across varying time horizons by setting *HORIZON* to 0 (diagnosis) or to a value greater than 0 (prediction). Predictor values from the baseline visit only were retained and copied forward to the rows corresponding to later visits, while the visit-specific target value (*DEMENTED*) and the elapsed time from baseline (*HORIZON*) were left unchanged. Thus, each retained row represents a patient-horizon observation based on baseline predictors and an observed follow-up time, rather than an independent patient. For model development, the target value was defined only for observed visits. Patients who had not developed dementia and were not followed to a future horizon were not assigned an outcome beyond their observed follow-up. Accordingly, the flexible-horizon ML training dataset did not generate additional labelled rows beyond observed visits. To make follow-up coverage transparent, we report the row-level HORIZON distribution and a patient-level known/censored-status summary in the Supplementary Materials (Supplemental Tables 4 and 5, respectively).

**Stage 3**: Rows and columns with more than 70% missing values were removed. This step resulted in the deletion of 1,799 rows and 36 variables, including, for example, all measures derived from the Montreal Cognitive Assessment (MoCA).

**Stage 4**: Highly correlated variables (correlation coefficient > 0.7) were excluded to minimise multicollinearity and information redundancy. Specifically, we removed six variables: *MMSEORDA*, *MMSEORLO*, *RIGDLOLF*, *RIGDUPLF*, *TRESTLHD* and *HXSTROKE*. At the end of this stage, 66 variables remained, which were carried forward for the modelling phase.

**Stage 5**: Recursive Feature Elimination (RFE) was applied as part of the model hyperparameter tuning process using a nested model selection pipeline (described below in Sect. 2.3). RFE was performed with a step size of 0.1, eliminating the 10% least important variables in each iteration. The number of variables to select was treated as a tuneable hyperparameter, with possible values ranging from 2 to 20 in increments of 2, and was optimised alongside the other model hyperparameters.

### Model Development

Six ML algorithms were considered: Logistic Regression, L2 Logistic Regression, KNN, Decision Tree, Random Forest and XGBoost. In addition, Cox Proportional Hazards model was included to provide survival analysis baselines against which the performance of our best flexible-horizon ML approach could be compared. Below, we first describe the data processing steps and the 5 × 2 nested cross-validation procedure for hyperparameter tuning and evaluation for the ML models, before outlining the training and evaluation procedures for the survival analysis model later in Sect. 2.5.

#### Data Processing and Model Pipeline

To prepare the dataset for modelling, missing values were imputed using the median for continuous and ordinal variables, and the mode for nominal variables. For the three non-tree-based algorithms (Logistic Regression, L2 Logistic Regression and KNN), features were standardised by subtracting the mean and dividing by the standard deviation, and nominal features were one-hot encoded within the model pipeline. Because these steps are not essential for tree-based models, they were omitted for Decision Tree, Random Forest, and XGBoost.

Since the non-dementia cases are substantially more prevalent than the dementia cases in the dataset, we employed SMOTE-NC (Synthetic Minority Over-sampling Technique for Nominal and Continuous) based on KNN [26] to address class imbalance. SMOTE-NC was applied only within training data: within each inner training fold during hyperparameter tuning, and within each outer training partition when refitting the selected model before evaluation on the held-out outer fold. Before applying SMOTE-NC, missing values were imputed using parameters estimated from the relevant training partition only. To preserve the flexible-horizon structure, SMOTE-NC was applied separately within each horizon group (i.e., for each distinct *HORIZON* value). If a horizon group contained only one outcome class or too few minority-class samples for nearest neighbour synthesis, that group was retained unchanged rather than oversampled. The resampled horizon groups were then concatenated and shuffled before model fitting. As an additional robustness check, we conducted a no-SMOTE-NC sensitivity analysis for the final KNN model using the same grouped held-out evaluation framework; this analysis showed a clear overall performance advantage for SMOTE-NC across evaluated horizons, particularly in terms of sensitivity, and is reported in the Supplementary Materials (see Supplemental Table 11).

For patients diagnosed with dementia at their first visit, all subsequent visit data was omitted from the analysis, and only their first visit data was kept. This decision was made because these patients would automatically be classified as having dementia in all subsequent time horizons, thereby potentially biasing the models especially during evaluation. After this final data preprocessing step, 134,117 samples remained for the modelling stage. Rows recorded after a participant’s first incident dementia diagnosis were retained in the model-development dataset. Among 3,986 participants with an incident dementia record, 2,376 contributed at least one subsequent row, giving 5,673 post-diagnosis rows.

#### Nested Cross-Validation and Hyperparameter Tuning

**Fig. 1:**
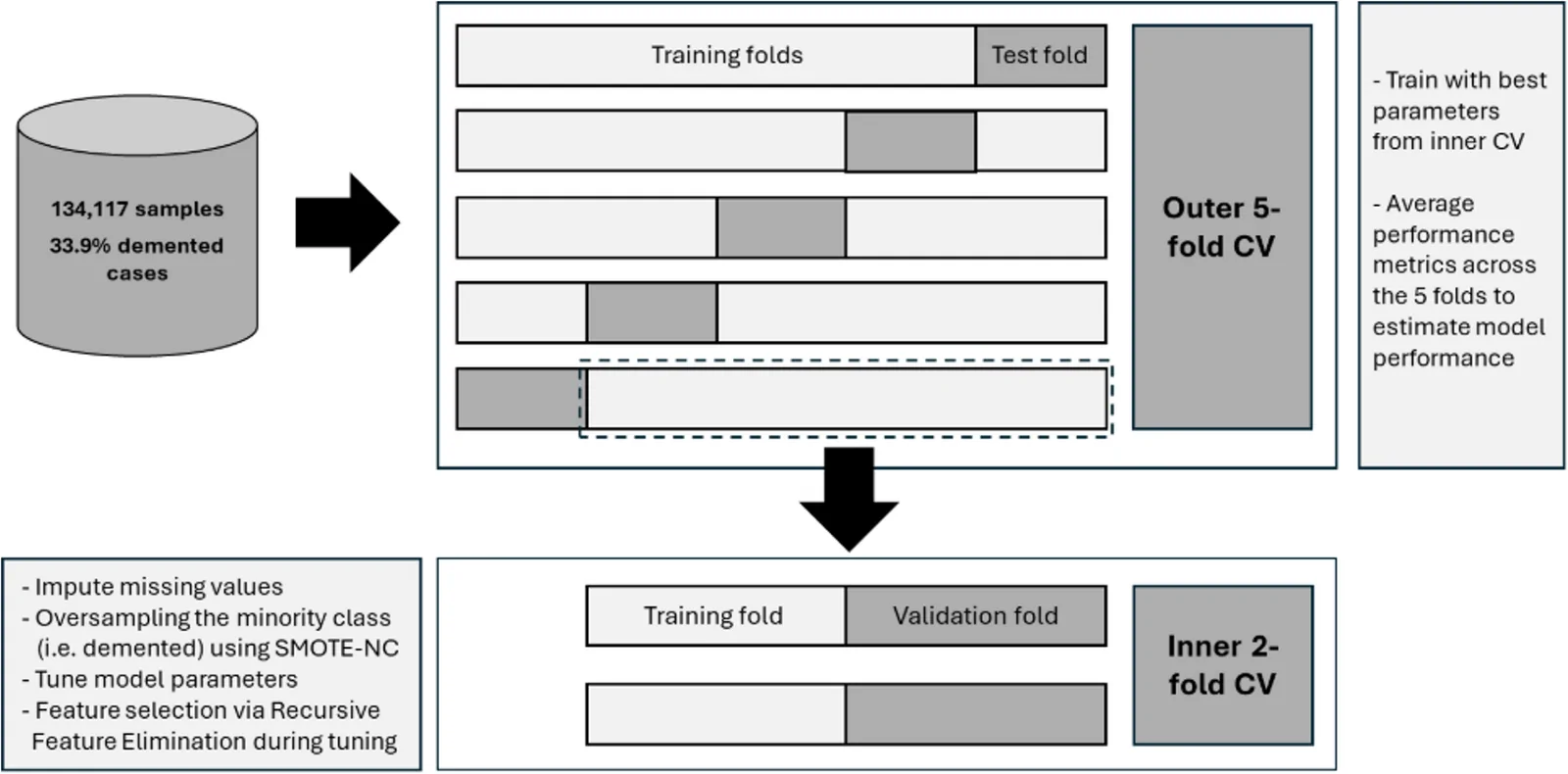
Overview of the nested 5 × 2 cross-validation (CV) framework used for model development and evaluation. The preprocessed dataset was divided into five outer folds to assess final performance, while each training fold was further split into two inner folds for hyperparameter tuning. The optimised model from the inner loop CV was retrained on the training fold and then evaluated on the held-out test fold in the outer loop. This process was repeated five times after which the performance metrics were averaged to estimate the model’s performance metrics. Imputation, scaling, one-hot encoding, SMOTE-NC and RFE were fitted only within training folds. The held-out outer test folds were used only for final performance estimation

We employed a 5 × 2 nested cross-validation framework (i.e., with 5-fold cross-validation in the outer loop and 2-fold cross-validation in the inner loop; [27]) for robust hyperparameter tuning and performance estimation (see Fig. 1 for a summary of the model development process). First, the dataset was split into five outer folds using stratified grouping by patient ID to ensure that no data from the same patient was used simultaneously for training and testing. Within each outer loop, the training data was further split to two inner folds to perform hyperparameter tuning via randomised grid search from specified hyperparameter ranges of up to 1,000 hyperparameter combinations. For each model, we tested a range of relevant hyperparameters (e.g., regularisation strength for L2 Logistic Regression, tree depth for Random Forest, number of neighbours for KNN, etc.). The exact list of parameter values is provided in Supplemental Table 2. All preprocessing and model selection steps, including imputation, scaling, one-hot encoding, SMOTE-NC and RFE, were performed within the training data of the relevant cross-validation split and did not use information from the held-out outer test folds.

We selected the best hyperparameter combination for each model based on the geometric mean (G-mean) of sensitivity and specificity (*G-mean*$\:=\sqrt{sensitivity\:\times\:specificity}$). Sensitivity (also known as recall) is defined as the proportion of true positive cases—that is, individuals with dementia who are correctly identified. Specificity is the proportion of true negative cases—i.e., those without dementia who are correctly classified as such. By taking the G-mean of sensitivity and specificity, we favoured models that balanced both metrics. After identifying the best hyperparameters in the inner loop, each model was retrained on the full training partition of the outer loop. The held-out outer fold was then used to estimate generalisation performance. This process was repeated across all five outer folds.

### Model Evaluation and Interpretation

#### Performance Evaluation Metrics

During model development, hyperparameter tuning and comparison of the candidate machine learning algorithms were conducted using patient-horizon rows generated by the flexible-horizon construction. Specifically, performance was estimated using the held-out outer folds from the nested cross-validation procedure described above. We evaluated model performance separately for same-visit diagnostic classification and future risk prediction. Same-visit diagnostic performance was evaluated using held-out rows with HORIZON = 0. Future prediction performance was evaluated at cumulative target horizons of 24, 48, 72, 96 and 120 months. For each target horizon greater than 0, performance was calculated using observed held-out patient-horizon rows satisfying 0 < HORIZON ≤ the target horizon. Thus, for example, the 48-month evaluation included all observed follow-up rows after baseline and up to 48 months, and an individual patient could contribute more than one row. These cumulative observed-row results were used to compare the candidate algorithms and select the best-performing model, and are reported in Supplemental Fig. 1. The cumulative evaluation was also used to assess fairness across protected features, which is described later in Sect.  2.4.3.

After the best model had been selected, its performance was evaluated at patient level for same-visit classification and future prediction. Same-visit classification used one baseline record per patient with HORIZON = 0. Future prediction was evaluated at the exact target horizons of 24, 48, 72, 96 and 120 months among the patients who were dementia-free at baseline, with one evaluation record created per eligible patient. At each target horizon *T*, a patient was classified as a case if dementia was first recorded by *T*; as a known non-case if dementia had not been recorded by *T* and a dementia-negative assessment was available at or after *T*; and as unknown/censored if follow-up ended before *T* without a recorded dementia diagnosis. A patient first recorded as dementia-positive after *T*, without a dementia-negative assessment at or after *T*, was also classified as unknown at *T* because the timing of conversion relative to the target horizon could not be determined. Performance calculations were based on cases and known non-cases. A fold-specific Kaplan–Meier inverse probability of censoring weighting (IPCW) analysis of area under the receiver operating characteristic curve (AUC) and Brier score is reported in Supplementary materials within the “Sensitivity analyses” section (Supplemental Table 10).

For each model, binary predictions and model risk scores from the held-out outer folds were used to calculate various performance metrics, including sensitivity, specificity, accuracy and AUC, as described below. 95% confidence intervals were obtained from 1000 patient-level bootstrap replicates, where patients were sampled with replacement within held-out outer folds, and all fixed horizons belonging to each sampled patients were retained in the same replicate. For transparency, cumulative observed-row performance of the selected KNN model is retained as a secondary analysis in Supplemental Table 12, together with a sensitivity analysis where performance was recalculated using patient-level weighting so that each patient contributed equal total weight within each held-out fold and evaluation horizon (see Supplemental Table 13).

##### Sensitivity:

Proportion of actual positive cases that are correctly identified as positive.

*Sensitivity=TP/(TP+FN)*

where *TP* (True Positives) refers to the number of actual dementia cases correctly identified as dementia and *FN* (False Negatives) is the number of actual dementia cases incorrectly identified as non-dementia.

##### Specificity:

Proportion of actual negative cases that are correctly identified as negative.

*Specificity=TN/(TN+FP)*

where *TN* (True Negatives) refers to the number of actual non-dementia cases correctly identified as such and *FP* (False Positives) is the number of actual non-dementia cases incorrectly identified as dementia.

##### Accuracy:

Proportion of all correctly classified cases (both positive and negative) among the total number of cases.

$$Accuracy=(TP+TN)\;/\;(TP+TN+FP+FN)$$

##### Area Under the Receiver Operating Characteristic Curve (AUC):

AUC score measures how well the model distinguishes between the positive and negative classes across various classification thresholds. An AUC score of 1 indicates perfect separability, whereas a score of 0.5 represents performance at chance level. A higher AUC score reflects better separability between the classes.

To choose the best-performing ML model, we again used the *G-mean* of sensitivity and specificity. Accuracy was reported for completeness, but was not used for model selection or as the primary basis for interpreting model performance. For each metric, results were calculated within each held-out outer fold and then averaged across the five outer folds.

Calibration of the final selected model (KNN) was assessed using the pooled cross-fitted predictions from the same-visit and future horizon patient-level evaluations described above. At each horizon, we calculated the observed event proportion (prevalence), mean model-estimated score, Brier score, AUC, calibration intercept and calibration slope and constructed quantile-based calibration plot. Calibration intercept and slope were estimated by fitting a logistic calibration model in which the observed outcome was regressed on the logit-transformed predicted model probability, with ideal calibration corresponding to an intercept of 0 and a slope of 1. These analyses describe calibration of the raw model scores, and no recalibration model was fitted.

We also calculated positive predictive value (PPV), negative predictive value (NPV), sensitivity, specificity, G-mean and confusion-matrix counts at illustrative raw model cutoffs of 0.10, 0.20 and 0.50. The cutoff of 0.50 corresponds to the default KNN majority-vote classification threshold used in the main binary performance analysis. The lower cutoffs are presented only to show operating-characteristic trade-offs and are not proposed as calibrated probability thresholds or deployment thresholds.

#### Model Explainability

We employed the SHapley Additive exPlanations (SHAP) technique [28] to provide feature-level explanations of model predictions. SHAP assigns each feature a contribution value (positive or negative) for each prediction, facilitating both global (feature importance) and local (i.e., case-by-case) interpretation. Performance estimation was completed using the nested cross-validation procedure described above. After this evaluation, a final KNN model was trained on the full analytic dataset using the best hyperparameters across all 10 inner folds for interpretation and prototype deployment. SHAP analysis was then applied post hoc to this final model, using the Permutation explainer with a background sample of 200 randomly selected data points from the full dataset and a randomly selected subset of 3,000 samples for explanation. SHAP analysis was conducted only after model development for post hoc interpretation and was not used for feature selection, hyperparameter tuning, model training or performance estimation.

#### Fairness Assessment

In addition to evaluating the overall predictive performance of our models, we conducted an exploratory assessment of how the performance of the selected ML model varied across three protected features: *SEX*, *RACE* and *AGE*. We computed the performance metrics—sensitivity, specificity, AUC, accuracy and the geometric mean of sensitivity and specificity (hereafter G-mean)—separately for each subgroup. More specifically, we considered:

- *SEX*: “Male” vs. “Female”.
- *RACE*: “White”, “Black or African American”, “Asian”, “American Indian or Alaska Native” and “Other” (including Native Hawaiian and Other Specific Pacific Islander).
- *AGE*: Three age ranges: “18–44”, “45–65” and “> 65”.

To quantify fairness, we used two commonly studied fairness metrics (equal opportunity and predictive equality) and introduced a third (G-mean equality), all based on the key performance measures used for model selection and evaluation. These were computed per outer fold and then averaged over the five outer cross-validation folds:

1. *Equal opportunity*: The ratio of true positive rates (i.e., sensitivity) between each comparison subgroup and the reference subgroup.
2. *Predictive equality*: The ratio of false positive rates (i.e., 1-specificity) between each comparison subgroup and the reference subgroup.
3. *G-mean equality*: The ratio of G-means between each comparison subgroup and the reference subgroup.

We used Female, White and > 65 years as reference groups for SEX, RACE and AGE, respectively, because these were the largest groups within each protected feature and therefore provided the most stable comparators for ratio-based subgroup metrics. We use the term reference group rather than privileged group to avoid implying that these categories are necessarily socially or clinically privileged in this dataset. In an ideal scenario, each fairness metric should be close to 1, though a commonly used threshold is 80% (i.e., a ratio between 0.80 and 1.25) [29, 30]. The fairness analysis was based on the observed patient-horizon row evaluation: subgroup metrics and ratios were calculated within each held-out outer fold and then averaged across folds. To support cautious interpretation, we also report 95% confidence intervals for subgroup performance metrics based on 1000 patient-level bootstrap replicates, as before. In addition, we report subgroup row counts, unique-patient counts and dementia-event counts in Supplemental Table 7.

### Comparison with Survival Analysis Modelling

To benchmark our best flexible-horizon ML approach against a standard time-to-event method, we implemented a Cox Proportional Hazards survival model using the same cohort, baseline predictor set (i.e. starting with the same 66 predictors later reduced with the RFE feature selection approach) and outer fold assignments. The Cox model used one baseline row per patient. Survival time was defined as the number of months from the first visit to the first visit at which dementia was recorded, while patients who did not develop dementia during follow-up were treated as right-censored at their last available visit. Model tuning for the Cox model was based on the concordance index (C-index), which is a standard discrimination metric for survival analysis because it evaluates the ranking of predicted time-to-event risk without requiring an arbitrary classification threshold.

For performance comparison, KNN and Cox were evaluated at 24, 48, 72, 96 and 120 months using the same patients and the same recorded dementia-by-horizon outcome definition described in Sect. 2.4.1. Each patient thus contributed one cross-fitted KNN risk score and one cross-fitted Cox cumulative-risk estimate at each horizon. Sensitivity, specificity and G-mean were the central comparative metrics because they directly quantify the operating trade-off emphasised in the KNN model-development procedure. Accuracy, AUC, Brier score and calibration intercept and slope were also calculated as complementary measures. Similarly to KNN evaluation, confidence intervals were obtained from 1,000 paired patient-level bootstrap replicates.

### Model Deployment

The interactive web application was built with Streamlit v1.44.1 as a research-only prototype to demonstrate the flexible-horizon model, support clinician feedback and facilitate future validation work. It is not intended for autonomous diagnosis, direct patient management or unsupervised clinical use. The intended users of the current version are the research team and other clinicians who can test model behaviour using historical or hypothetical patient profiles. The application provides model-derived dementia classification or risk prediction, together with explanatory outputs, to support clinician judgement; it does not replace clinical assessment or independently establish a dementia diagnosis.

Currently, the public demo (https://dementiaapp.aezzizi.com/) runs on an Amazon Web Services EC2 t2.micro instance (1 vCPU, 1 GB RAM and 16GB SSD for storage) under Ubuntu 22.04 LTS. Access control is enforced through password protection (see Notes). No user data are retained after the browsing session, and all predictions are computed locally on the EC2 instance. The prototype has not yet undergone formal cybersecurity assessment, clinical safety review, regulatory assessment or usability testing. Any future clinical implementation would require more restrictive access control, audit logging, information-governance review, version control for model updates, external validation and recalibration, and compliance with relevant regulatory requirements. The Supporting Materials include a brief illustrative guide to using the application (see Supplemental Figs. 4 and 5).

### Apparatus

All analyses except for the hyperparameter grid search were conducted on a Windows 11 laptop equipped with 4 CPU cores, 32GB RAM and an NVIDIA Geforce GTX 1660 Ti. The grid search was performed on a high-performance computing server with 128 CPU cores and an NVIDIA A100 GPU, allowing faster and more extensive model training. Our main computational environment was Python 3.11, with key libraries including pandas, xgboost, imbalanced-learn, sklearnex (scikit-learn with Intel optimisations), scikit-survival for survival analysis modelling, and SHAP for explainability. 2

## Results

### Patient Characteristics

Following the data preparation process, the final analytic sample included a total of 134,117 visit records from 47,017 unique patients. On average, each patient had 2.9 visits (range: 1–18), with just over half (54.1%) having only a single visit (for a more detailed patient row contribution summary, see Supplemental Table 3). Among all patients, 33.9% were diagnosed with dementia at their first visit. Table 1 summarises the baseline characteristics of the patients by dementia status at their initial visit.

**Table 1:** Baseline characteristics by dementia status

Characteristic	No dementia	Dementia
Participants, No. (%)	31,079 (66.1%)	15,938 (33.9%)
Age, mean in years (SD)	71.2 (10.4)	72.9 (10.5)
Sex		
Men, No. (%)	12,519 (40.3%)	7,681 (48.2%)
Women, No. (%)	18,560 (59.7%)	8,257 (51.8%)
Race		
White, No. (%)	24,302 (78.7%)	13,299 (84.1%)
Black or African American, No. (%)	4,989 (16.2%)	1,650 (10.4%)
American Indian or Alaska Native, No. (%)	269 (0.9%)	147 (0.9%)
Native Hawaiian or Other Pacific, No. (%)	24 (0.1%)	24 (0.2%)
Asian, No. (%)	899 (2.9%)	343 (2.2%)
Other, No. (%)	405 (1.3%)	351 (2.2%)
Education, mean in years (SD)	15.5 (3.2)	14.4 (3.7)
Independence level		
Independent, No. (%)	27,907 (90.1%)	3,151 (20.0%)
Requires some assistance, No. (%)	2,601 (8.4%)	7,264 (46.1%)
Requires basic assistance, No. (%)	409 (1.3%)	3,694 (23.5%)
Completely dependent, No. (%)	43 (0.1%)	1,641 (10.4%)
BMI, mean (SD)	27.5 (5.5)	26.4 (5.0)
Total MMSE^1^ score, mean (SD)	28.2 (2.2)	19.8 (6.9)
Animal naming test, mean (SD)	18.9 (5.9)	10.2 (5.4)
GDS^2^ score, mean (SD)	1.9 (2.4)	2.9 (2.9)

^1^ MMSE: Mini-Mental State Examination; ^2^ GDS: Geriatric Depression Scale

Patients without dementia were slightly younger on average (71.2 years) than those with dementia (72.9 years). Women constituted 59.7% of the non-dementia group versus 51.8% in the dementia group. Overall, 80.5% of patients were White (78.7% of those without dementia and 84.1% of those with dementia), whereas Black or African American patients represented 14.2% of the total cohort (16.2% of the non-dementia and 10.4% of the dementia group). The other racial categories combined accounted for the remaining 5.3% of the patients. Educational attainment was generally higher among subjects without dementia (mean [SD]: 15.5 [3.2] years) compared with those with dementia (14.4 [3.7] years). In terms of functional independence, 90.1% of the non-dementia group was fully independent at baseline, whereas only 20.0% of the dementia group maintained full independence. Most patients with dementia required some level of assistance (46.1%), and 10.4% were completely dependent. Cognitive assessment measures aligned strongly with dementia status: The mean [SD] total MMSE score was 28.2 [2.2] among individuals not affected by dementia compared to 19.8 [6.9] in patients with dementia. The animal naming test followed a similar pattern (18.9 [5.9] vs. 10.2 [5.4]), while the GDS score was higher in those with dementia (2.9 [2.9] vs. 1.9 [2.4]).

The observed *HORIZON* distribution is summarised in Supplemental Table 4. Same-visit classification rows accounted for 35.1% of the records, while future prediction rows were distributed across 1–24 months (17.8%), 25–48 months (18.2%), 49–72 months (12.0%), 73–96 months (7.5%), 97–120 months (4.6%), and beyond 120 months (4.7%). The corresponding row-level dementia prevalence values were 33.9%, 7.7%, 11.3%, 12.1%, 11.4%, 11.6% and 9.6%, respectively. Among the 31,079 patients who were dementia-free at baseline, recorded dementia status was ascertainable for 16,973 (54.6%), 12,447 (40.0%), 9,513 (30.6%), 7,532 (24.2%) and 6,214 (20.0%) at 24, 48, 72, 96 and 120 months, respectively (Supplemental Table 5).

### Model Performance

Performance comparisons across the six candidate ML algorithms revealed that the K-Nearest Neighbour (KNN) model achieved the highest geometric mean (G-mean) of sensitivity and specificity over the five outer cross-validation loops (mean [SD]: 0.837 [0.002]; see Supplemental Fig. 1). Random Forest followed closely (0.834 [0.003]) with a notably lower sensitivity (0.805 [0.011] vs. 0.867 [0.012] for KNN) and higher specificity (0.865 [0.009] vs. 0.809 [0.007]). In secondary care settings—where patients are referred due to suspected cases of dementia—maximising sensitivity takes priority to ensure that fewer actual dementia cases go undetected. Thus, the stronger sensitivity of KNN offers a notable advantage as all other candidate models had sensitivities below 0.8. The nested cross-validation procedure identified a consistent set of optimal hyperparameters for KNN with Euclidean distance: 18 or 20 selected features, 100 neighbours and uniform weighting (see Supplemental Table 2 for the complete results of the hyperparameter tuning process). This means that in practice, this final KNN model makes a classification by comparing each patient to the 100 most similar patients with known dementia status—based on the 20 features described in Table 2—and weighing each of the similar patients equally.

**Table 2:** List of variables used in the best performing machine learning model (KNN)

Variable	Description	Predictor category
HORIZON	Time horizon for the dementia prediction (in months)	Model-derived (horizon) variable
AGE	Subject’s age at baseline visit (in months).	Pre-diagnostic risk factor
EDUC	Years of education.	Pre-diagnostic risk factor
NACCLIVS [With partner]	1 if living with a spouse or partner; 0 otherwise.	Pre-diagnostic risk factor
NACCBMI	Body Mass Index (BMI).	Pre-diagnostic (modifiable) risk factor
BPSYS	Sitting systolic blood pressure.	Pre-diagnostic (modifiable) risk factor
HACHIN	Hachinski ischemic score.	Clinical examination finding
NACCGDS	Total score on the Geriatric Depression Scale (GDS).	Clinical examination finding
RIGDUPRT	Rigidity in the right upper extremity (scored 0 = none to 4 = severe).	Clinical examination finding
BRADYKIN	Body bradykinesia and hypokinesia (scored 0 = none to 4 = marked movement impairment).	Clinical examination finding
ANIMALS	Total number of animals named in 60 s.	Cognitive test measure
NACCMMSE	Total Score on the Mini-Mental State Examination (MMSE).	Cognitive test measure
INDEPEND	Level of independence (scored 1 = able to live independently to 4 = completely dependent).	Functional symptom measure
BILLS [Normal]	Binary indicator: 1 if the patient was able to perform financial tasks, such as writing checks, paying bills or balancing chequebooks as they normally would in the past four weeks; 0 otherwise.	Functional symptom measure
EVENTS [Normal]	1 if the subject was able to keep track of current events as they normally would in the past four weeks; 0 otherwise.	Functional symptom measure
PAYATTN [Normal]	1 if the subject was able to pay attention and understand media, such as TV programmes, books or magazines as they normally would in the past four weeks; 0 otherwise.	Functional symptom measure
REMDATES [Normal]	1 if the subject was able to remember appointments, family occasions, holidays or medications as they normally would in the past four weeks; 0 otherwise.	Functional symptom measure
TRAVEL [Normal]	1 if the subject was able to travel out of the neighbourhood, drive or arrange to take public transportation as they normally would in the past four weeks; 0 otherwise.	Functional symptom measure
TAXES [Normal]	1 if the subject was able to assemble tax records, business affairs or other papers as they normally would in the past four weeks; 0 otherwise.	Functional symptom measure
TAXES [Dependent]	1 if the subject depended completely on others to assemble tax records, business affairs or other papers in the past four weeks; 0 otherwise.	Functional symptom measure

Table 3 reports the patient-level performance of the final KNN model. Same-visit classification (*Horizon* = 0) achieved sensitivity of 0.951 (95% CI 0.947–0.954), specificity of 0.810 (0.805–0.814) and G-mean of 0.877 (0.874–0.880). Across the evaluated future horizons, sensitivity remained consistently high, ranging from 0.791 to 0.833. At 24 months, sensitivity was 0.808 (0.789–0.827), specificity was 0.856 (0.850–0.861) and G-mean was 0.831 (0.822–0.842). At 120 months, the corresponding values were 0.833 (0.822–0.845), 0.706 (0.689–0.724) and 0.767 (0.756–0.778). The results show that the flexible-horizon KNN framework retained strong sensitivity across all evaluated horizons, with the expected change in the sensitivity-specificity balance as the prediction horizon increased.

**Table 3:** Patient-level performance of the final KNN model for same-visit classification and fixed-horizon future prediction. For each future horizon, one cross-fitted prediction was generated per baseline dementia-free patient and performance was evaluated among patients whose recorded dementia status by that horizon was ascertainable. Values are point estimates (95% patient-bootstrap confidence intervals; 1,000 replicates)

Evaluation	Horizon (months)	Sensitivity	Specificity	G-mean	AUC	Accuracy
Same-visit diagnosis	0	0.951 (0.947, 0.954)	0.810 (0.805, 0.814)	0.877 (0.874, 0.880)	0.959 (0.957, 0.960)	0.857 (0.854, 0.860)
Incident- dementia prediction	24	0.808 (0.789, 0.827)	0.856 (0.850, 0.861)	0.831 (0.822, 0.842)	0.906 (0.899, 0.913)	0.852 (0.846, 0.857)
	48	0.791 (0.775, 0.805)	0.838 (0.831, 0.845)	0.814 (0.806, 0.822)	0.893 (0.886, 0.899)	0.828 (0.821, 0.834)
	72	0.800 (0.786, 0.813)	0.795 (0.785, 0.806)	0.797 (0.789, 0.806)	0.877 (0.870, 0.885)	0.797 (0.788, 0.805)
	96	0.824 (0.811, 0.836)	0.748 (0.734, 0.762)	0.785 (0.776, 0.794)	0.863 (0.855, 0.872)	0.784 (0.775, 0.793)
	120	0.833 (0.822, 0.845)	0.706 (0.689, 0.724)	0.767 (0.756, 0.778)	0.851 (0.842, 0.862)	0.784 (0.774, 0.794)

Calibration results are summarised in Table 4 and Supplemental Fig. 2. Same-visit classification had an observed event proportion of 0.339, a mean KNN risk score of 0.436, a Brier score of 0.097, a calibration intercept of -1.225 and a calibration slope of 1.108. Calibration varied across future horizons. At 24 months, the observed event proportion was 0.093 and the mean KNN risk score was 0.239, with a calibration intercept of -1.774 and slope of 0.944. The difference between the observed event proportion and mean score narrowed at 96 months (0.476 versus 0.497), while at 120 months the observed event proportion was 0.609 and the mean score was 0.556, with an intercept of 0.270 and slope of 1.280. Thus, the calibration pattern changed systematically across the evaluated horizon range rather than showing uniform overestimation.

**Table 4:** Calibration summary for the final KNN model for same-visit classification and future prediction at the specified horizons. The table reports observed event proportion, mean model-estimated risk score, Brier score, AUC, calibration intercept and calibration slope

Evaluation	Horizon	Observed prevalence	Mean model-estimated risk score	Brier score	AUC	Calibration intercept	Calibration slope
Same-visit diagnosis	0	0.339	0.436	0.097	0.959	-1.225	1.108
Incident- dementia prediction	24	0.093	0.239	0.102	0.906	-1.774	0.944
	48	0.220	0.327	0.120	0.893	-0.923	0.994
	72	0.345	0.419	0.140	0.877	-0.514	1.040
	96	0.476	0.497	0.152	0.863	-0.145	1.128
	120	0.609	0.556	0.156	0.851	0.270	1.280

Threshold-based analyses further showed that predictive values depended strongly on the chosen threshold and evaluation horizon. At the default KNN threshold 0.50, same-visit classification had PPV = 0.719 and NPV = 0.970. For future prediction horizons, PPV increased from 0.365 at 24 months to 0.815 at 120 months, while NPV changed from 0.977 to 0.731 at 120 months. At lower illustrative thresholds of 0.10 and 0.20, sensitivity increased but specificity and PPV decreased, as expected. Full confusion matrix counts and operating characteristics are provided in Supplemental Table 8 under the “Calibration and additional model evaluation” section. These cutoffs are illustrative raw-score cutoffs and are not proposed as calibrated probability thresholds or clinical deployment thresholds.

**Fig. 2:**
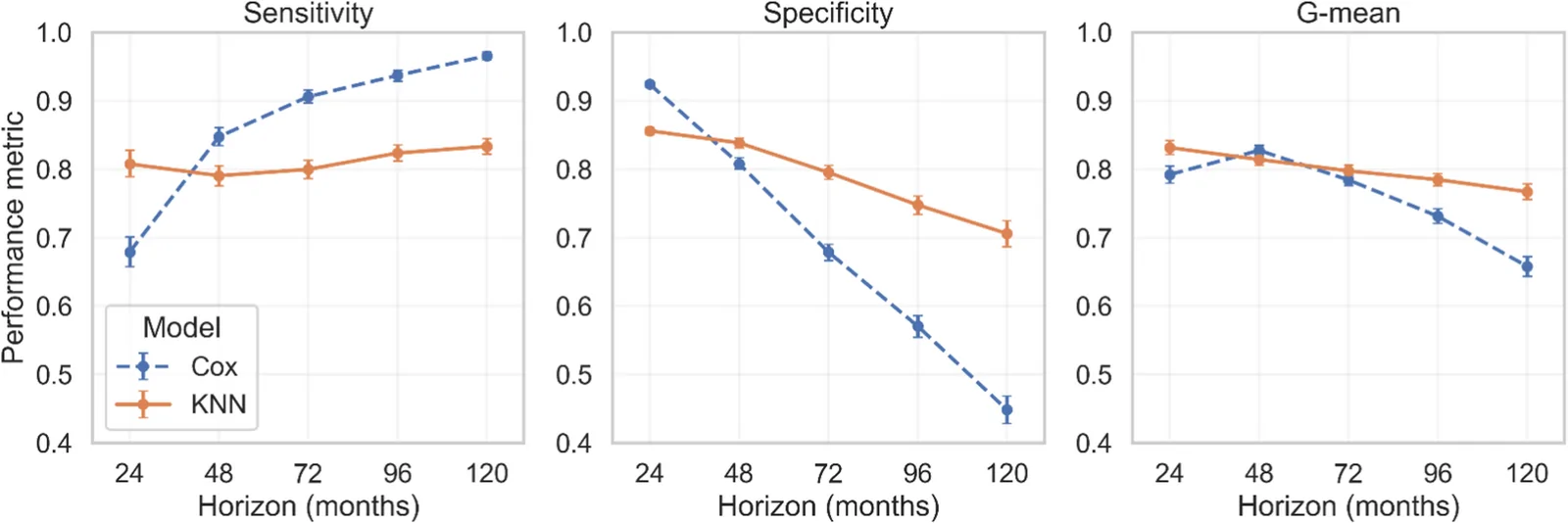
Comparison of KNN and Cox proportional hazards model performance across prediction horizons (24, 48, 72, 96, and 120 months). Sensitivity, specificity, and G-mean are shown for each model, with orange solid lines representing KNN and blue dashed lines representing the Cox model. Error bars denote the corresponding patient-level bootstrap 95% confidence intervals computed based on 1000 replicates

Finally, to contextualise the flexible-horizon ML approach, we compared our KNN model with a Cox proportional hazards model on the common subset of non-zero horizons (i.e., excluding *HORIZON* = 0, which is not supported by the survival model; the optimal hyperparameter sets across the five outer folds for the survival model are listed in Supplemental Table 6). The comparative results are presented in Fig. 2. At 24 months, KNN achieved higher sensitivity (0.808 [95% CI 0.789–0.827] versus 0.679 [0.658–0.701]) and G-mean (0.831 [0.822–0.842] versus 0.792 [0.779–0.805]), while Cox achieved higher specificity (0.924 [0.920–0.928] versus 0.856 [0.851–0.861]). At 48 months, Cox had higher sensitivity (0.847 [0.834–0.861] versus 0.791 [0.776–0.805]), KNN had higher specificity (0.838 [0.831–0.846] versus 0.808 [0.800-0.817]), and Cox had a slightly higher G-mean (0.827 [0.820–0.835] versus 0.814 [0.814 [0.805–0.823]). From 72 to 120 months, Cox achieved higher sensitivity (between 0.906 and 0.966 versus 0.800-0.833), whereas KNN retained substantially higher specificity (0.795 − 0.706 versus 0.679 − 0.449) and higher G-mean (0.797 − 0.767 versus 0.784 − 0.658). Paired KNN-minus-Cox G-mean differences (where the same sampled patients were used to compute 95% confidence intervals) were 0.039 (95% CI 0.028–0.050), -0.013 (-0.020 to -0.006), 0.013 (0.005–0.020), 0.053 (0.044–0.063) and 0.109 (0.095–0.124) at 24, 48, 72, 96 and 120 months, respectively. Therefore, KNN achieved the higher G-mean at four of five horizons, while the two models offered complementary sensitivity-specificity profiles. Comprehensive discrimination, prediction-error and calibration comparative results are provided in Supplemental Tables 9 and Supplemental Fig. 3.

### Model Explainability

**Fig. 3:**
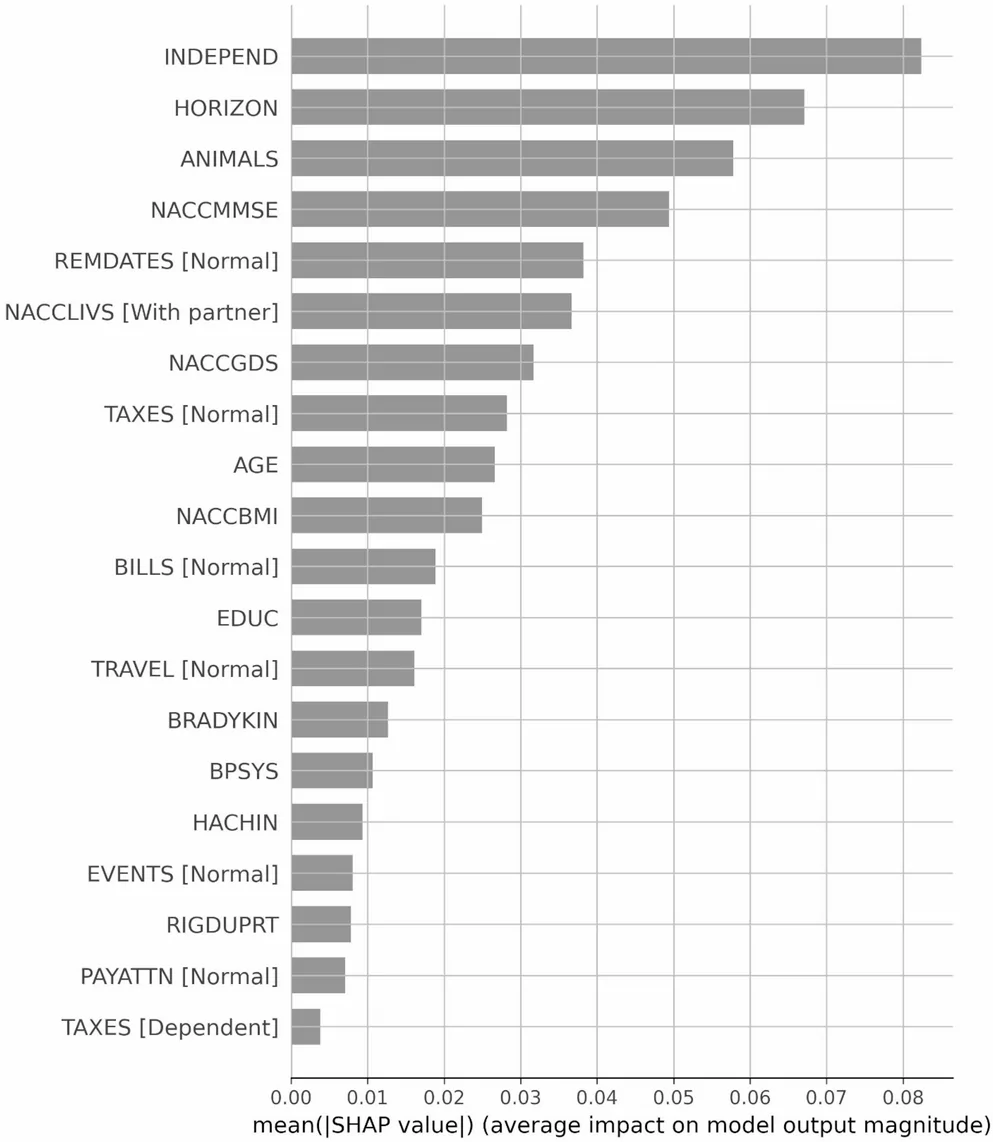
SHAP-based feature importance ranking for the KNN model. Features are ranked in descending order based on their mean absolute SHAP values, which indicate their relative contribution to the model’s predictions

To understand how each feature contributed to the KNN model’s predictions, we conducted a variable impact analysis based on the SHapley Additive exPlanations (SHAP) technique. Figure 3 ranks the 20 selected predictors by their mean absolute SHAP values, revealing that the indicator of functional independence (*INDEPEND*) and the prediction horizon (*HORIZON*) are the two most influential features. Cognitive assessment variables—particularly the animal naming test (*ANIMALS*), MMSE (*NACCMMSE*) and geriatric depression scale (*NACCGDS*) scores—also rank highly, highlighting their strong discriminative power for differentiating dementia from non-dementia. Furthermore, simple indicators of memory for dates (*REMDATES*), living with a partner (*NACCLIVS [With partner]*) and ability to assemble tax records (*TAXES [Normal]*) can provide useful and easy-to-obtain predictive information about dementia status. Interestingly, Age appears lower on the ranking than might be expected, likely because its effect is partially absorbed by *HORIZON* (e.g., a 10-year horizon would typically imply that the patient is older at the time of diagnosis relative to a 2-year horizon).

**Fig. 4:**
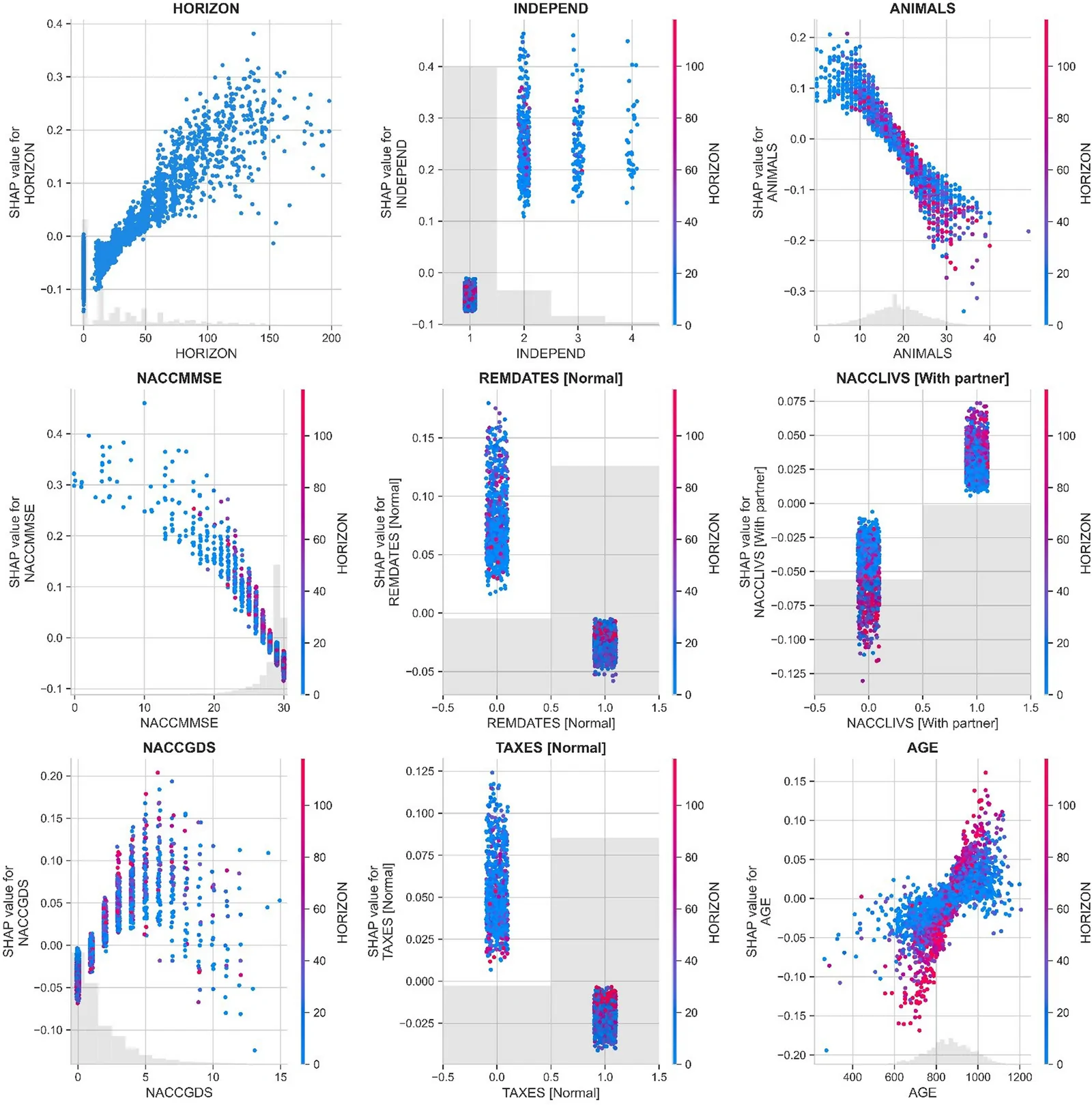
SHAP dependence plots showing the (interaction) effects of prediction horizon (top left) and the next eight top features. The x-axis shows feature values, and the y-axis shows each feature’s SHAP contribution. Data points correspond to visit samples and are colour-coded by the horizon value (blue for shorter horizons and red for longer horizons). *HORIZON* and *AGE* values are given in months

Next, we examined the effect of *HORIZON* on dementia classification, along with its interaction with the next eight most important features (Fig. 4). Each dot in the dependence graphs represents one visit sample. A positive SHAP value indicates that the feature increases the model-estimated dementia risk score, whereas a negative value suggests a pull towards a non-dementia classification. The model-estimated risk score of dementia increases with increasing SHAP values. For the interaction dependence graphs, points are shaded along a blue–red gradient corresponding to the *HORIZON* value (blue for shorter horizons, red for longer horizons).

In the *HORIZON* dependence plot, SHAP values increase with longer prediction windows, suggesting that a greater time interval from the baseline visit inclines the model towards predicting dementia. Age shows particularly strong interaction with *HORIZON*; older age raises the likelihood of dementia prediction, but the effect is even more pronounced when combined with longer prediction windows (indicated by the steeper slope for the red points). The remaining features exhibit expected clinical patterns without substantial horizon-specific interactions. For example, lower ANIMALS (animal-naming test score), lower NACCMMSE (MMSE test score) and higher NACCGDS (geriatric depression scale score) all increase the model-estimated dementia risk, consistent with well-established risk factors. Other features such as INDEPEND (functional independence), REMDATES (ability to remember important dates of significance to the subject), TAXES (ability to assemble taxes or business affairs) and NACCLIVS (living status) display generally consistent effects on dementia classification across different time horizons. More specifically, patients needing assistance (INDEPEND > 1), reporting poor date recall in the last 28 days (*REMDATES [NORMAL] = False*), reporting abnormal ability to assemble tax records or business affairs in the last 28 days (*TAXES [NORMAL] = False*), or living with a partner (*NACCLIVS [With partner] = True*) are predicted to be at increased risk. The latter effect is possibly reflecting that individuals living alone are likely to maintain greater independence, thus potentially mitigating dementia risk.

**Fig. 5:**
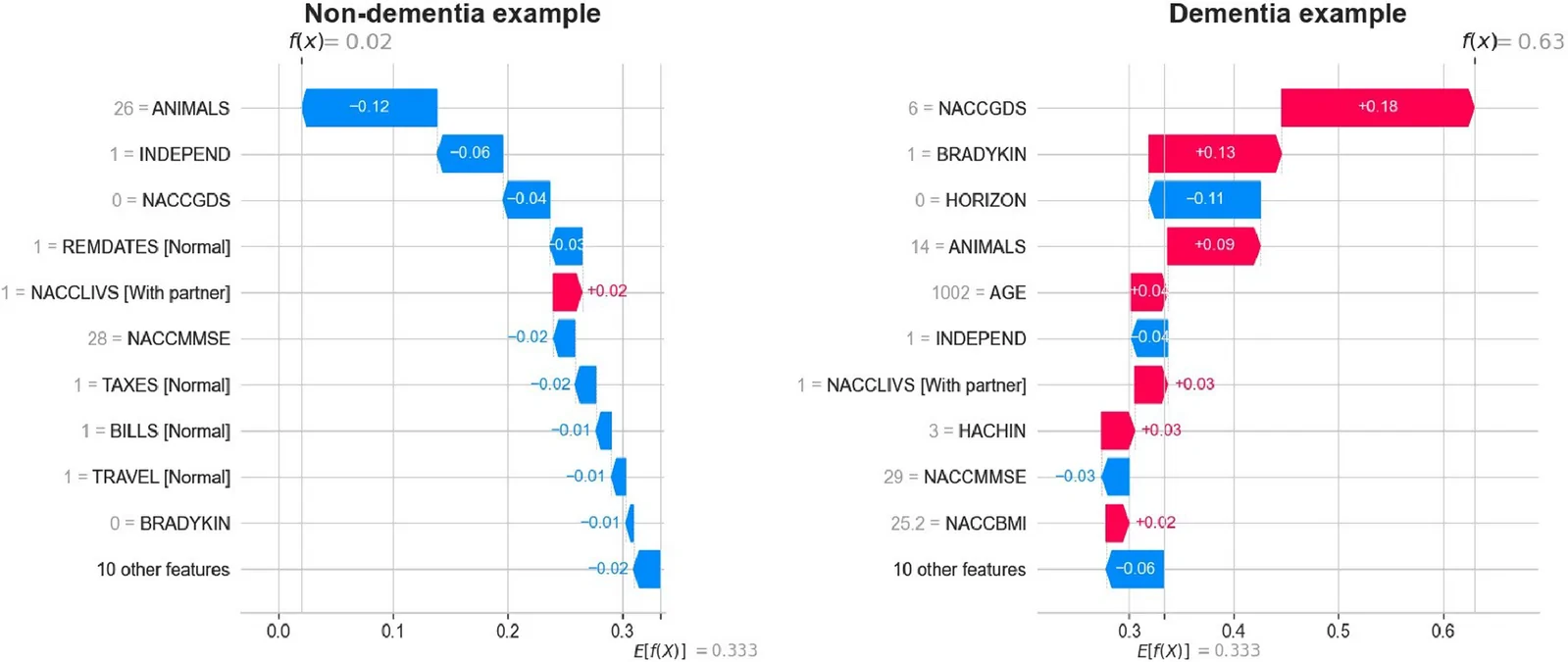
SHAP waterfall plots for two individual predictions: a non-dementia case (left) and a dementia case (right). Each bar shows how a specific feature’s value shifts the model-derived dementia risk score up (red) or down (blue). The sum of these shifts, plus the base value (*E[f(x)]*) produces the final model-derived risk score *f(x)*

While these global explanations provide a valuable overview of which features steer our model’s predictions and how they do so, local explanation—focusing on individual patient predictions—are equally valuable to enhance the reliability and transparency of the model, which in turn would promote its adoption for clinical use. Figure 5 illustrates such local explanations via two SHAP “waterfall” plots: one for a randomly selected non-dementia case (left panel) and one for a dementia case (right panel). The horizontal axis represents SHAP values, with an initial “base value” that is common to all samples (akin to an intercept in regression). Red bars extending to the right indicates that feature’s value increases the model-estimated dementia risk, whereas blue bars extending to the left reduce it.

In the non-dementia example, the patient’s high animal-naming score (*ANIMALS* = 26) strongly lowers the model-estimated dementia risk score, while the reported living with a partner (*NACCLIVS [With partner] = True*) slightly raises it. Overall, most features shift the score towards a negative diagnosis. By contrast, in the dementia example, the geriatric depression scale score (*NACCGDS* = 6) and reported body bradykinesia/hypokinesia (minimal slowness reported for this patient) exerts the largest positive pull towards a dementia diagnosis, ultimately bringing the final model-estimated dementia risk score to 0.63.

### Fairness Evaluation

In an exploratory analysis, we examined how the performance of the KNN model varied across subgroups defined by *SEX*, *RACE* and *AGE* (Table 5), while recognising that some subgroup estimates are based on relatively small sample and event counts (see Supplemental Table 7). Table 5 shows that G-mean and AUC score remained consistently high across most subgroups, indicating that classification performance (balancing sensitivity and specificity) is relatively uniform. There are, however, notable differences in sensitivity vs. specificity within specific subgroups. Among *SEX* groups, males have higher sensitivity than females (0.886 [95% CI: 0.877–0.894] vs. 0.849 [0.840–0.858]) but lower specificity (0.743 [0.735–0.751] vs. 0.851 [0.846–0.856]). Across racial subgroups, G-means were relatively close, ranging from 0.812 (0.780–0.842) in the “Other” race group to 0.860 (0.822–0.896) in the “American Indian or Alaska Native” group. However, the “Asian” and “Other” race subgroups showed higher sensitivity (0.928 [0.894–0.956] and 0.935 [0.904–0.964], respectively) but lower specificity (0.755 [0.725–0.784] and 0.708 [0.662–0.759], respectively) compared with the “White” subgroup, which had a sensitivity of 0.867 (0.861–0.874) and specificity of 0.803 (0.797–0.808). *AGE*-based stratification shows strong performance for the “45–65” and “>65” groups, whereas the smaller “18–44” group has a lower sensitivity (0.678 [0.571–0.816]) but very high specificity (0.901 [0.865–0.935]).

**Table 5:** Observed-row performance of the final KNN model across subgroups of the protected features. Values are reported as mean (95% confidence interval). Confidence intervals were obtained from 1,000 patient-level bootstrap replicates

Protected feature	Subgroup	Sensitivity	Specificity	G-mean	ROC AUC	Accuracy
Sex	Female	0.849 (0.840, 0.858)	0.851 (0.846, 0.856)	0.850 (0.845, 0.855)	0.930 (0.927, 0.934)	0.851 (0.846, 0.855)
	Male	0.886 (0.877, 0.894)	0.743 (0.735, 0.751)	0.811 (0.806, 0.817)	0.905 (0.901, 0.909)	0.775 (0.769, 0.782)
Race	White	0.867 (0.861, 0.874)	0.803 (0.797, 0.808)	0.834 (0.830, 0.838)	0.918 (0.915, 0.921)	0.815 (0.811, 0.820)
	Black or African American	0.835 (0.811, 0.855)	0.856 (0.846, 0.867)	0.845 (0.834, 0.857)	0.931 (0.923, 0.938)	0.853 (0.844, 0.862)
	Asian	0.928 (0.894, 0.956)	0.755 (0.725, 0.784)	0.837 (0.816, 0.857)	0.929 (0.914, 0.943)	0.785 (0.762, 0.809)
	American Indian or Alaska Native	0.858 (0.804, 0.915)	0.865 (0.819, 0.902)	0.860 (0.822, 0.896)	0.939 (0.914, 0.960)	0.864 (0.827, 0.896)
	Other	0.935 (0.904, 0.964)	0.708 (0.662, 0.759)	0.812 (0.780, 0.842)	0.927 (0.906, 0.946)	0.782 (0.750, 0.816)
Age	> 65	0.866 (0.860, 0.873)	0.788 (0.782, 0.793)	0.826 (0.822, 0.830)	0.913 (0.910, 0.916)	0.803 (0.798, 0.807)
	45–65	0.876 (0.862, 0.889)	0.883 (0.874, 0.891)	0.879 (0.871, 0.887)	0.948 (0.942, 0.953)	0.882 (0.874, 0.889)
	18–44	0.678 (0.571, 0.816)	0.901 (0.865, 0.935)	0.780 (0.708, 0.854)	0.921 (0.893, 0.953)	0.872 (0.835, 0.909)

Table 6 summarises the pairwise fairness metrics: *equal opportunity* (the ratio of each subgroup’s true positive rate to that of the reference subgroup), *predictive equality* (the ratio of false positive rates) and *G-mean equality* (the ratio of G-means). Ratios below or above the commonly cited 0.80–1.25 range indicate potential disparity. In terms of *equal opportunity*, most subgroups were close to 1, suggesting no severe disadvantage in correctly identifying dementia cases, except that the youngest group (18–44) showed a lower true positive ratio (0.783 [95% CI: 0.657–0.942]). *Predictive equality* exhibited the largest deviations, with several comparisons (Male vs. Female, Other vs. White, etc.) outside the commonly referenced 0.80–1.25 range; in some cases, the non-reference group had a higher false positive rate (e.g., males, Asian), whereas in others (e.g., Black or African American) it was lower than that of the reference subgroup. *G‐mean* ratios were closer to 1, ranging from 0.945 (0.858–1.034) for the 18–44 age group to 1.065 (1.054–1.075) for the 45–65 age group. Thus, the overall balance of sensitivity and specificity was broadly similar across most groups, despite differences in the individual operating characteristics.

**Table 6:** Comparison of observed-row fairness metrics across subgroups of the protected features. Values are reported as mean (95% confidence interval). Confidence intervals were obtained from 1,000 patient-level bootstrap replicates. Ratios below or above the commonly cited 0.80–1.25 range indicate potential disparity

Protected feature	Reference subgroup	Comparison subgroup	Equal opportunity	Predictive equality	G-mean equality
Sex	Female	Male	1.044 (1.028, 1.059)	1.733 (1.655, 1.820)	0.955 (0.946, 0.963)
Race	White	Black or African American	0.963 (0.934, 0.988)	0.728 (0.673, 0.782)	1.013 (0.998, 1.027)
		Asian	1.069 (1.031, 1.101)	1.240 (1.090, 1.405)	1.003 (0.978, 1.027)
		American Indian or Alaska Native	0.989 (0.925, 1.056)	0.679 (0.488, 0.912)	1.031 (0.985, 1.072)
		Other	1.078 (1.040, 1.110)	1.480 (1.219, 1.723)	0.973 (0.934, 1.008)
Age	> 65	45–65	1.011 (0.993, 1.028)	0.549 (0.509, 0.593)	1.065 (1.054, 1.075)
		18–44	0.783 (0.657, 0.942)	0.468 (0.305, 0.639)	0.945 (0.858, 1.034)

Taken together, the exploratory subgroup analysis showed broadly consistent balanced discrimination across most groups, while also identifying differences in the sensitivity–specificity trade-off. In addition, subgroup calibration was not assessed given that the raw KNN outputs were not calibrated as absolute probabilities and several subgroup-by-outcome combinations were too sparse to support stable calibration estimates. The current results should therefore be viewed as an initial internal fairness assessment rather than evidence that the model is equitable across all demographic groups.

## Discussion

In this study, we present a novel ML-based solution that can both diagnose dementia and predict its onset over flexible time horizons with high sensitivity and specificity, by focusing only on 20 routinely collected secondary care variables. In doing so, our work addresses two major gaps in the existing literature: (1) the inability of previously developed ML models to simultaneously detect current dementia status and predict its onset over varying future horizons; and (2) the challenge of balancing robust performance with important responsible AI principles of interpretability and fairness. Besides, an online version of the developed model is available at https://dementiaapp.aezzizi.com/.^3^

### Summary of Findings

Our KNN-based model achieved the strongest performance among the six candidate ML algorithms, with robust internally validated discrimination. Same-visit classification achieved sensitivity of 0.951 and G-mean of 0.877, while sensitivity remained between 0.791 and 0.833 across future horizons from 24 to 120 months. These findings demonstrate the feasibility of using a single clinically informed model for both current dementia classification and future risk prediction over a flexible user-specified horizon, using 20 routinely collected secondary-care variables. By contrast, most earlier models reported either suboptimal sensitivity or specificity, and were restricted to a single fixed prediction horizon [13–15].

When compared on the common non-zero horizon subset, our flexible-horizon KNN model achieved competitive performance with that of a Cox survival model, while offering the additional advantages of supporting (zero-horizon) point-of-diagnosis classification and being implementable as a standard supervised ML pipeline without the extra assumptions and setup required for survival modelling. More specifically, Cox prioritised sensitivity increasingly strongly at longer horizons, whereas KNN retained substantially greater specificity, high sensitivity levels (> 0.79) and achieved the higher G-mean at four of the five evaluated horizons.

Our model also compares favourably with previously published studies, across several prediction horizons, including at diagnostic time point [31], 12-month horizon [14], 29 months [13], and even far longer span of 144 months [15] (for a more detailed comparison, refer to Supplemental Table 14). These cross-study comparisons are useful for situating our results within the published literature, although they should not be regarded as conclusive evidence of superior performance, since the reported results differ in cohort composition, feature sets and validation procedures.

KNN’s instance-based mechanism appears to be especially well suited for this task. By comparing a target patient’s profile with a pool of 100 similar profiles, it effectively mimics a clinical reasoning process wherein decisions are guided by a wealth of prior experience diagnosing similar cases. Crucially, because *HORIZON* is treated as just another feature (rather than a cutoff), the model can learn from subgroups that have different lengths of follow-up and extrapolate across them accordingly, within the represented range of the training data. Other features with clinical importance, like cognitive assessment scores, can then be weighted more heavily when *HORIZON* is less relevant for a particular patient, or vice versa. These factors likely explain why the proposed model maintained high performance across a wide spectrum of prediction horizons.

### Explainability and Fairness Considerations

Emerging consensus in dementia research highlights the importance of interpretable ML approaches for promoting clinician trust and adoption [32–34]. Our approach incorporates global and local explainability using SHAP. Globally, we show that *HORIZON* is the second most influential feature, indicating that a greater time lag from baseline is associated with an elevated risk of dementia. Scores from well-established cognitive tests (e.g., MMSE, animal-naming test) and markers of independence contributed heavily to classification, aligning with standard clinical understanding. On a patient-by-patient basis, we illustrate how local SHAP analysis can be used to identify the specific factors driving an individual’s classification.

Equally important for healthcare applications is ensuring ML model performance equity across (protected) groups [35, 36] (or at least, clearly defining the boundaries of the model applicability). Our exploratory fairness analysis adds a substantive responsible AI dimension by examining sensitivity, specificity and their balance across sex, race and age subgroups. G-mean remained broadly similar across most groups, supporting the overall stability of balanced discrimination, while differences in sensitivity and specificity identified distinct false-negative and false-positive trade-offs. For example, males and individuals categorised as “Other” race showed lower specificity despite higher sensitivity, while the 18–44 group showed lower sensitivity than older age groups. These differences may have different clinical consequences: false negatives could delay further assessment and support, whereas false positives could increase anxiety and unnecessary referral burden. Importantly, some subgroup estimates were based on relatively small sample and event counts, so the absence of a strong signal in this internal analysis should not be interpreted as proof of equity across all subgroups. Future external validation should therefore assess discrimination, subgroup calibration, false-positive and false-negative patterns and clinical consequences within sufficiently powered demographic subgroups before deployment. Should improving specificity or sensitivity for specific subgroups become necessary, bias-correction techniques could be applied during model development, though such adjustments might compromise overall model performance [37].

### Clinical Implications

In the UK’s NHS and other healthcare systems, long waiting times, restricted accessibility to dementia clinics and the need to attend multiple clinic appointments remain significant barriers to timely dementia diagnosis [5]. The model’s 20 inputs were deliberately selected for compatibility with secondary-care workflows. Many consist of routinely recorded demographic and physical measures or short questions about functional independence. The principal structured assessments are also relatively brief: the MMSE generally requires approximately 5–10 min [38], the short-form GDS approximately 5–7 min [39], and the animal-naming task uses a 60-second response period [40]. The remaining inputs include the Hachinski ischaemic score, brief neurological observations, blood pressure and body mass index, some of which may already be available from the clinical record.

On this basis, we hypothesise that collection or retrieval of the required inputs and generation of the model output could be completed in around 30 min within a suitably prepared secondary-care workflow. If confirmed prospectively, this could represent a meaningful reduction in focused data collection time relative to a conventional memory clinic appointment, which typically takes 1.5 h or more [41], while allowing specialist time to be concentrated on clinical interpretation and more complex cases. The 30-minute estimate was not measured in the present study and should therefore be tested through a prospective timed workflow and usability study. Besides, the model is intended to complement, rather than replace, comprehensive clinical assessment.

Importantly, the integration of modifiable risk factors (e.g., body mass index, blood pressure, depression measures) within the model allows for future prevention-oriented interventions [42] (although further adaptations maybe needed). For example, if the model forecasts a high dementia risk, clinicians could simulate how modifying specific risk factors, based on local SHAP outputs, might alter the outcome before determining a care plan. Ideally, the model would be integrated into existing digital medical workflows, or even better, run automatically during routine checks, similar to the processes already in place for diabetes risk assessment in the UK [43].

### Limitations and Future Work

Despite these promising results, several limitations merit discussion. First, our model combines all-cause dementia into a single classification label, overlooking distinctions among Alzheimer’s disease, vascular dementia and other dementia-related disease types. Future research could develop ML models with more granular diagnostic categories [44], and potentially, extend the “all-in-one” concept to differentiate dementia subtypes across varying prediction horizons. Second, while our final feature set is well suited for secondary care settings, it may be inappropriate for primary care, where certain variables—such as MMSE or formal depression scores—are not routinely assessed. Developing a parallel model tailored for general practitioner settings is a logical next step. Such model would rely on a subset of the accessible and easy-to-measure variables that we considered in this study (e.g., blood pressure measurements, body mass index, simple indicators of functional independence).

Third, although we evaluated same-visit diagnostic performance and future prediction performance separately, the present study did not train separate task-specific diagnostic and prognostic models. This was intentional because the aim was to test a single flexible-horizon framework, but future work should compare the unified approach against separate models optimised specifically for same-visit detection and incident risk prediction. Fourth, all training and validation data originate from the NACC UDS. Whereas this dataset is large and well curated, it is not based on a purely random sample, as participants are recruited mainly through referrals. As such, it may not fully represent patient demographics, referral pathways and assessment procedures in other healthcare systems, including UK NHS secondary care. Accordingly, the model should not be considered clinically ready at this stage. Before any real-world use in patient management, it requires external validation in independent cohorts, recalibration where necessary, prospective testing, clinician usability evaluation, assessment of workflow impact, and review against relevant clinical governance and regulatory requirements.

The flexible-horizon training construction allows patients with more observed visits to contribute more patient-horizon rows, including 5,673 rows recorded after the first incident dementia diagnosis. Grouped nested cross-validation prevented patient-level leakage, and the patient-level future-horizon evaluation avoided repeated counting when estimating reported performance. Nevertheless, unequal contribution during model fitting may still influence the fitted classifier. In addition, recorded outcome status was ascertainable for a decreasing proportion of the baseline (dementia-free) cohort at longer horizons, so selective follow-up may influence long horizon estimates. The IPCW analysis and the secondary observed-row and patient-weighting analyses in Supplemental Tables 10, 12 and 13 provide complementary robustness checks. Together, these analyses consistently support strong discrimination across the evaluated horizons, while external validation in cohorts with systematic follow-up remains important.

A further limitation concerns calibration of the raw KNN score. Although the final KNN model achieved strong discrimination, the post hoc calibration analysis showed that its raw neighbour-proportion outputs were not fully calibrated as absolute probabilities, particularly for shorter horizons (≤ 72). Predicted probabilities tended to overestimate observed event proportions across shorter horizons. Accordingly, the current model outputs should be interpreted as model-estimated risk scores rather than externally validated calibrated probabilities. Future work should evaluate recalibration methods, such as Platt scaling, isotonic regression or calibration-in-the-large [45], within independent secondary-care cohorts before any threshold-based clinical use.

Finally, we compared the flexible-horizon ML model with a Cox proportional hazards model as a conventional and widely used survival-analysis benchmark. However, other time-to-event approaches, including discrete-time survival models [46], pooled logistic regression [47], random survival forests and gradient-boosted survival models [48], may provide closer or complementary comparisons to the proposed horizon-conditioned framework. Evaluating these approaches in external datasets is an important methodological direction for future work, but was beyond the scope of the present study.

Our planned next step is a staged validation and translation pathway. First, we will retrospectively evaluate the model using historical data from the clinician co-author’s memory clinic service and assess whether recalibration is required. Second, if retrospective performance is acceptable, we will conduct silent prospective testing in which model outputs are generated but not used for patient management. Third, we will evaluate usability with clinicians and assess whether the outputs are understandable, actionable and appropriately calibrated to clinical workflows. Fourth, we will assess workflow impact, including potential effects on referral prioritisation, assessment burden, false positives and false negatives. Finally, any move toward implementation would require clinical governance review, cybersecurity assessment, regulatory review, and integration into existing electronic clinical systems if performance and safety are confirmed. Throughout this pathway, the model would remain a decision-support tool intended to inform clinician judgement rather than replace clinical assessment.

## Conclusion

In conclusion, this study provides proof-of-concept evidence that a clinically informed flexible-horizon machine learning framework can support both same-visit dementia classification and future incident dementia prediction within a single decision-support model. By combining routinely collected secondary care variables with model explainability and fairness assessment, the proposed approach addresses key practical and responsible AI considerations relevant to eventual clinical translation. Future work should focus on external validation, recalibration, prospective testing, usability evaluation and governance review before any use in clinical decision making or workflow implementation.

## Supplementary Information

Below is the link to the electronic supplementary material.

Supplementary Material 1

Supplementary Material 2

## Data Availability

The NACC UDS dataset is publicly available upon request from the National Alzheimer’s Coordinating Center (https://naccdata.org/).

## Footnotes

1 Access to the web application is currently password-protected and can be provided to clinicians upon request. The Supporting Materials also include abrief illustrative guide to using the application.

2 Reporting was guided by the TRIPOD + AI statement [49]. The completed checklist is provided as a separate supplementary file.

3 Access to the web application is currently password-protected and can be provided to clinicians upon request. The Supporting Materials also include abrief illustrative guide to using the application.

## Code Availability

The code used to reproduce the analyses will be made available on GitHub at https://github.com/Adnane017/Flexible_horizon_dementia_prediction_with_responsible_AI. The NACC UDS data cannot be redistributed by the authors and must be requested directly from NACC under their data-use procedures.
