# Supplementary material for: A Flexible-Horizon Clinical Decision Support Model for Dementia Early Detection and Risk Prediction in Secondary Care: A Responsible AI Approach
Source: J Med Syst. 2026 Sep 15;50(1):132. doi: 10.1007/s10916-026-02458-2 (PMC13578025; doi:10.1007/s10916-026-02458-2)
Supplement: Supplementary file 1 [file 10916_2026_2458_MOESM1_ESM.docx]

# **Supplemental Materials**

**Supplemental Table 1.** List of NHS conform variables and whether they were retained for machine learning modelling**.**

|  | **Category** | **Variable** | **Description** | **Retained (Y/N)** |
| --- | --- | --- | --- | --- |
| 1 | Subject Demographics | SEX | Subject’s sex | Y |
| 2 |  | EDUC | Years of education | Y |
| 3 |  | NACCLIVS | Living situation | Y |
| 4 |  | INDEPEND | Level of independence | Y |
| 5 |  | NACCAGE | Subject’s age at visit (converted to age in months) | Y |
| 6 | Subject Family History | NACCFAM | Indicator of first-degree family member with cognitive impairment | Y |
| 7 |  | NACCMOM | Indicator of mother with cognitive impairment | Y |
| 8 |  | NACCDAD | Indicator of father with cognitive impairment | Y |
| 9 | Subject Medications | ANYMEDS | Subject taking any medications | Y |
| 10 |  | NACCADEP | Reported current use of an antidepressant | Y |
| 11 |  | NACCAPSY | Reported current use of an antipsychotic agent | Y |
| 12 |  | NACCAANX | Reported current use of an anxiolytic, sedative, or hypnotic agent | Y |
| 13 | Subject Health History | ALCFREQ | During the past three months, how often did the subject have at least one drink of any alcoholic beverage such as wine, beer, malt liquor, or spirits? | N |
| 14 |  | CVHATT | Heart attack/cardiac arrest | Y |
| 15 |  | HATTMULT | More than one heart attack/cardiac arrest? | N |
| 16 |  | HATTYEAR | Year of most recent heart attack | N |
| 17 |  | CVAFIB | Atrial fibrillation | Y |
| 18 |  | CVANGIO | Angioplasty/endarterectomy/stent | Y |
| 19 |  | CVBYPASS | Cardiac bypass procedure | Y |
| 20 |  | CVPACDEF | Pacemaker and/or defibrillator | N |
| 21 |  | CVPACE | Pacemaker | Y |
| 22 |  | CVCHF | Congestive heart failure | Y |
| 23 |  | CVANGINA | Angina | N |
| 24 |  | CVHVALVE | Heart valve replacement or repair | N |
| 25 |  | CVOTHR | Other cardiovascular disease | Y |
| 26 |  | CBSTROKE | Stroke | Y |
| 27 |  | STROKMUL | More than one stroke reported as of the Initial Visit | N |
| 28 |  | ALCOHOL | Alcohol abuse — clinically significant occurring over a 12-month period manifested in one of the following areas: work, driving, legal, or social | Y |
| 29 |  | NACCBMI | Body mass index (BMI) | Y |
| 30 | Blood Pressure Addendum | BPSYSL | Subject blood pressure (sitting), systolic | Y |
| 31 | HIS and CVD | STEPWISE | Stepwise deterioration (re: cognitive status) | Y |
| 32 |  | HXSTROKE | History of stroke | N |
| 33 |  | FOCLSYM | Focal neurological symptoms | Y |
| 34 |  | FOCLSIGN | Focal neurological signs | Y |
| 35 |  | HACHIN | Hachinski ischemic score | Y |
| 36 | Unified Parkinson’s Disease Rating Scale (UPDRS) | TRESTFAC | Tremor at rest — face, lips, chin | Y |
| 37 |  | TRESTRHD | Tremor at rest — right hand | Y |
| 38 |  | TRESTLHD | Tremor at rest — left hand | N |
| 39 |  | TRESTRFT | Tremor at rest — right foot | Y |
| 40 |  | TRESTLFT | Tremor at rest — left foot | Y |
| 41 |  | RIGDNECK | Rigidity — neck | Y |
| 42 |  | RIGDUPRT | Rigidity — right upper extremity | Y |
| 43 |  | RIGDUPLF | Rigidity — left upper extremity | N |
| 44 |  | RIGDLORT | Rigidity — right lower extremity | Y |
| 45 |  | RIGDLOLF | Rigidity — left lower extremity | N |
| 46 |  | BRADYKIN | Body bradykinesia and hypokinesia | Y |
| 47 | Geriatric Depression Scale (GDS) | NOGDS | Is the subject able to complete the GDS, based on the clinician’s best judgment? | Y |
| 48 |  | SATIS | Are you basically satisfied with your life? | Y |
| 49 |  | DROPACT | Have you dropped many of your activities and interests? | Y |
| 50 |  | EMPTY | Do you feel that your life is empty? | Y |
| 51 |  | BORED | Do you often get bored? | Y |
| 52 |  | SPIRITS | Are you in good spirits most of the time? | Y |
| 53 |  | AFRAID | Are you afraid that something bad is going to happen to you? | Y |
| 54 |  | HAPPY | Do you feel happy most of the time? | Y |
| 55 |  | HELPLESS | Do you often feel helpless? | Y |
| 56 |  | STAYHOME | Do you prefer to stay at home, rather than going out and doing new things? | Y |
| 57 |  | MEMPROB | Do you feel you have more problems with memory than most? | Y |
| 58 |  | WONDRFUL | Do you think it is wonderful to be alive now? | Y |
| 59 |  | WRTHLESS | Do you feel pretty worthless the way you are now? | Y |
| 60 |  | ENERGY | Do you feel full of energy? | Y |
| 61 |  | HOPELESS | Do you feel that your situation is hopeless? | Y |
| 62 |  | BETTER | Do you think that most people are better off than you are? | Y |
| 63 |  | NACCGDS | Total GDS Score | Y |
| 64 | Functional Activities Questionnaire (FAQ) | BILLS | In the past four weeks, did the subject have any difficulty or need help with: Writing checks, paying bills, or balancing a checkbook | Y |
| 65 |  | TAXES | In the past four weeks, did the subject have any difficulty or need help with: Assembling tax records, business affairs, or other paper | Y |
| 66 |  | SHOPPING | In the past four weeks, did the subject have any difficulty or need help with: Shopping alone for clothes, household necessities, or groceries | Y |
| 67 |  | GAMES | In the past four weeks, did the subject have any difficulty or need help with: Playing a game of skill such as bridge or chess, working on a hobby | Y |
| 68 |  | STOVE | In the past four weeks, did the subject have any difficulty or need help with: Heating water, making a cup of coffee, turning off the stove | Y |
| 69 |  | MEALPREP | In the past four weeks, did the subject have any difficulty or need help with: Preparing a balanced meal | Y |
| 70 |  | EVENTS | In the past four weeks, did the subject have any difficulty or need help with: Keeping track of current events | Y |
| 71 |  | PAYATTN | In the past four weeks, did the subject have any difficulty or need help with: Paying attention to and understanding a TV program, book, or magazine | Y |
| 72 |  | REMDATES | In the past four weeks, did the subject have any difficulty or need help with: Remembering appointments, family occasions, holidays, medications | Y |
| 73 |  | TRAVEL | In the past four weeks, did the subject have any difficulty or need help with: Traveling out of the neighborhood, driving, or arranging to take public transportation | Y |
| 74 | Physical/Neurological Exam Findings | MMSECOMP | Was any part of the MMSE completed? | N |
| 75 |  | MMSEORDA | Orientation subscale score — Time | N |
| 76 |  | MMSEORLO | Orientation subscale score — Place | N |
| 77 |  | PENTAGON | Intersecting pentagon subscale score | Y |
| 78 |  | NACCMMSE | Total MMSE score (using D-L-R-O-W) | Y |
| 79 |  | ANIMALS | Animals — Total number of animals named in 60 seconds | Y |
| 80 |  | UDSVERFN | Number of F-words repeated in 1 minute | N |
| 81 | Neuropsychological Battery Scores | MOCACOMP | Was any part of MoCA administered? | N |
| 82 |  | MOCAREAS | If no part of MoCA administered, reason code | N |
| 83 |  | MOCATOTS | MoCA Total Raw Score — uncorrected | N |
| 84 |  | NACCMOCA | MoCA Total Score — corrected for education | N |
| 85 |  | MOCATRAI | MoCA: Visuospatial/executive — Trails | N |
| 86 |  | MOCACUBE | MoCA: Visuospatial/executive — Cube | N |
| 87 |  | MOCACLOC | MoCA: Visuospatial/executive — Clock contour | N |
| 88 |  | MOCACLON | MoCA: Visuospatial/executive — Clock numbers | N |
| 89 |  | MOCACLOH | MoCA: Visuospatial/executive — Clock hands | N |
| 90 |  | MOCANAMI | MoCA: Language — Naming | N |
| 91 |  | MOCAREGI | MoCA: Memory — Registration (two trials) | N |
| 92 |  | MOCADIGI | MoCA: Attention — Digits | N |
| 93 |  | MOCALETT | MoCA: Attention — Letter A | N |
| 94 |  | MOCASER7 | MoCA: Attention — Serial 7s | N |
| 95 |  | MOCAREPE | MoCA: Language — Repetition | N |
| 96 |  | MOCAFLUE | MoCA: Language — Fluency | N |
| 97 |  | MOCAABST | MoCA: Abstraction | N |
| 98 |  | MOCARECN | MoCA: Delayed recall — No cue | N |
| 99 |  | MOCARECC | MoCA: Delayed recall — Category cue | N |
| 100 |  | MOCARECR | MoCA: Delayed recall — Recognition | N |
| 101 |  | MOCAORDT | MoCA: Orientation — Date | N |
| 102 |  | MOCAORMO | MoCA: Orientation — Month | N |
| 103 |  | MOCAORYR | MoCA: Orientation — Year | N |
| 104 |  | MOCAORDY | MoCA: Orientation — Day | N |
| 105 |  | MOCAORPL | MoCA: Orientation — Place | N |
| 106 |  | MOCAORCT | MoCA: Orientation — City | N |
| 107 | Clinician Diagnosis | ALCABUSE | Alcohol abuse diagnosis | N |

**Supplemental Table 2.** Summary of the hyperparameter tuning process and results for machine learning models.

| **Machine learning algorithm** | **Hyperparameter range** | **Best hyperparameters across all 10 inner folds** | **Average inner CV g-mean of sensitivity and specificity** | **Outer fold** | **Best hyperparameters within outer fold** | **Average g-mean within outer fold** |
| --- | --- | --- | --- | --- | --- | --- |
| *Logistic Regression* | num_features: [2, 4, 6, 8, 10, 12, 14, 16, 18, 20] | num_features = 14 | 0.781 | 1 | num_features = 8 | 0.804 |
|  |  |  |  | 2 | num_features = 8 | 0.782 |
|  |  |  |  | 3 | num_features = 14 | 0.795 |
|  |  |  |  | 4 | num_features = 6 | 0.773 |
|  |  |  |  | 5 | num_features = 18 | 0.79 |
| *L2 Logistic Regression* | num_features: [2, 4, 6, 8, 10, 12, 14, 16, 18, 20];  C: [0.01, 0.1, 1, 10, 100] | num_features = 20;  C = 0.01 | 0.817 | 1 | num_features = 12; C = 0.01 | 0.82 |
|  |  |  |  | 2 | num_features = 12; C = 0.01 | 0.814 |
|  |  |  |  | 3 | num_features = 20; C = 0.01 | 0.818 |
|  |  |  |  | 4 | num_features = 16; C = 0.01 | 0.819 |
|  |  |  |  | 5 | num_features = 12; C = 0.01 | 0.82 |
| *KNN* | num_features: [2, 4, 6, 8, 10, 12, 14, 16, 18, 20];  n_neighbors: [5, 10, 25, 50, 100];  weights: ['uniform', 'distance'] | num_features = 20; n_neighbors = 100;  weights = 'uniform' | 0.837 | 1 | num_features = 18; n_neighbors = 100; weights = 'uniform' | 0.837 |
|  |  |  |  | 2 | num_features = 18; n_neighbors = 100; weights = 'uniform' | 0.836 |
|  |  |  |  | 3 | num_features = 20; n_neighbors = 100; weights = 'uniform' | 0.835 |
|  |  |  |  | 4 | num_features = 20; n_neighbors = 100; weights = 'uniform' | 0.839 |
|  |  |  |  | 5 | num_features = 20; n_neighbors = 100; weights = 'uniform' | 0.838 |
| *Decision Tree* | num_features: [2, 4, 6, 8, 10, 12, 14, 16, 18, 20];  max_depth: [2, 3, 4]; max_features: ['sqrt', 0.5, 0.75];  min_samples_split: [10, 50, 100, 500] | num_features = 4; max_depth = 3;  max_features = 'sqrt'; min_samples_split = 500 | 0.817 | 1 | num_features = 20; max_depth = 3; max_features = 0.5; min_samples_split = 500 | 0.821 |
|  |  |  |  | 2 | num_features = 20; max_depth = 3; max_features = 0.5; min_samples_split = 500 | 0.82 |
|  |  |  |  | 3 | num_features = 4; max_depth = 3; max_features = 'sqrt'; min_samples_split = 10 | 0.816 |
|  |  |  |  | 4 | num_features = 4; max_depth = 3; max_features = 'sqrt'; min_samples_split = 10 | 0.816 |
|  |  |  |  | 5 | num_features = 4; max_depth = 3; max_features = 'sqrt'; min_samples_split = 10 | 0.817 |
| *Random Forest* | num_features: [2, 4, 6, 8, 10, 12, 14, 16, 18, 20];  N_estimators: [25, 50, 100, 250, 500, 750, 1000];  max_depth: [2, 3, 4, 5, 6, 7, 8]; max_features: ['sqrt', 0.5, 0.75]; min_samples_split: [2, 5, 10] | num_features = 20; N_estimators = 500;  max_depth = 8; max_features = 'sqrt'; min_samples_split = 5 | 0.836 | 1 | num_features = 20; N_estimators = 500; max_depth = 8; max_features = 'sqrt'; min_samples_split = 5 | 0.838 |
|  |  |  |  | 2 | num_features = 20; N_estimators = 500; max_depth = 8; max_features = 'sqrt'; min_samples_split = 5 | 0.837 |
|  |  |  |  | 3 | num_features = 18; N_estimators = 25; max_depth = 8; max_features = 'sqrt'; min_samples_split = 10 | 0.836 |
|  |  |  |  | 4 | num_features = 20; N_estimators = 500; max_depth = 8; max_features = 'sqrt'; min_samples_split = 5 | 0.836 |
|  |  |  |  | 5 | num_features = 20; N_estimators = 1000; max_depth = 8; max_features = 'sqrt'; min_samples_split = 2 | 0.837 |
| *XGBoost* | num_features: [2, 4, 6, 8, 10, 12, 14, 16, 18, 20];  N_estimators: [25, 50, 75, 100, 250, 500];  max_depth: [2, 3, 4, 5, 6, 7, 8];  learning_rate: [0.005, 0.01, 0.025, 0.05, 0.1, 0.2] | num_features = 20; N_estimators = 50;  max_depth = 3;  learning_rate = 0.1 | 0.833 | 1 | num_features = 20; N_estimators = 50; max_depth = 3; learning_rate = 0.1 | 0.836 |
|  |  |  |  | 2 | num_features = 18; N_estimators = 75; max_depth = 3; learning_rate = 0.05 | 0.832 |
|  |  |  |  | 3 | num_features = 20; N_estimators = 100; max_depth = 3; learning_rate = 0.05 | 0.834 |
|  |  |  |  | 4 | num_features = 20; N_estimators = 50; max_depth = 3; learning_rate = 0.1 | 0.833 |
|  |  |  |  | 5 | num_features = 16; N_estimators = 75; max_depth = 4; learning_rate = 0.05 | 0.832 |

**Supplemental Table 3.** Patient row contribution summary.

| **measure** | **value** |
| --- | --- |
| Unique patients | 47,017 |
| Mean rows per patient | 2.85 |
| Median rows per patient | 1 |
| Patients with one row, n (%) | 25,453 (54.1%) |
| Maximum rows per patient | 18 |
| Mean maximum observed horizon (months) | 27.7 |
| Median maximum observed horizon (months) | 0 |
| IQR maximum observed horizon (months) | 0-43 |
| Maximum observed horizon (months) | 206 |
| Patients with >=24 months follow-up, n (%) | 17,229 (36.6%) |
| Patients with >=48 months follow-up, n (%) | 11,148 (23.7%) |
| Patients with >=72 months follow-up, n (%) | 7,120 (15.1%) |
| Patients with >=96 months follow-up, n (%) | 4,519 (9.6%) |
| Patients with >=120 months follow-up, n (%) | 2,743 (5.8%) |
| Patients with >120 months follow-up, n (%) | 2,636 (5.6%) |

**Supplemental Table 4.** Row-level distribution of observed *HORIZON* values in the final analytic modelling dataset. *HORIZON* = 0 corresponds to same-visit diagnostic classification. Future horizon rows correspond to observed follow-up visits only.

| **Horizon window** | **Rows** | **Row (%)** | **Unique patients** | **Dementia-positive rows** | **Non-dementia rows** | **Dementia prevalence (%)** |
| --- | --- | --- | --- | --- | --- | --- |
| 0 (same visit) | 47,017 | 35.1 | 47,017 | 15,938 | 31,079 | 33.9 |
| 1–24 months | 23,936 | 17.8 | 19,437 | 1,836 | 22,100 | 7.7 |
| 25–48 months | 24,414 | 18.2 | 15,747 | 2,762 | 21,652 | 11.3 |
| 49–72 months | 16,129 | 12.0 | 10,283 | 1,951 | 14,178 | 12.1 |
| 73–96 months | 10,113 | 7.5 | 6,462 | 1,157 | 8,956 | 11.4 |
| 97–120 months | 6,233 | 4.6 | 4,108 | 722 | 5,511 | 11.6 |
| >120 months | 6,273 | 4.7 | 2,636 | 601 | 5,672 | 9.6 |

**Supplemental Table 5.** Patient-level known, censored and dementia status at each target horizon at baseline. A patient was classified as a case if dementia was recorded by the target horizon, as a known non-case if a dementia-negative assessment was available at or after the target horizon, and as censored/ unknown otherwise. The calculations exclude patients who were diagnosed with dementia at baseline as done in our ML modelling for target horizons > 0.

| **Target horizon (months)** | **Unique patients** | **Known status by horizon** | **Dementia by horizon** | **Known non-dementia through horizon** | **Censored/unknown before horizon** | **Known status (%)** | **Dementia among known (%)^1^** |
| --- | --- | --- | --- | --- | --- | --- | --- |
| 24 | 31,079 | 16,973 | 1,580 | 15,393 | 14,106 | 54.6 | 9.3 |
| 48 | 31,079 | 12,447 | 2,740 | 9,707 | 18,632 | 40.0 | 22.0 |
| 72 | 31,079 | 9,513 | 3,286 | 6,227 | 21,566 | 30.6 | 34.5 |
| 96 | 31,079 | 7,532 | 3,588 | 3,944 | 23,547 | 24.2 | 47.6 |
| 120 | 31,079 | 6,214 | 3,786 | 2,428 | 24,865 | 20.0 | 60.9 |

^1^ The column “Dementia among known (%)” should be interpreted as the incident dementia proportion among patients with ascertainable status by the target horizon, not as population prevalence or cumulative incidence.

**Supplemental Figure 1.** Secondary cumulative observed row comparison of candidate machine learning algorithms. For each target horizon greater than zero, the analysis includes observed held-out patient-horizon rows satisfying 0 < HORIZON ≤ the stated target horizon (thus patients may contribute multiple rows). Error bars indicate 95% confidence intervals across the five outer folds.

**Supplemental Table 6.** Summary of the hyperparameter tuning process and results for the Cox Proportional Hazards model.

| **Hyperparameter range** | **Outer fold** | **Best hyperparameters within outer fold** | **Average C-index within outer fold** | **Average G-mean within outer fold** |
| --- | --- | --- | --- | --- |
| num_features: [2, 4, 6, 8, 10, 12, 14, 16, 18, 20];  alpha: [0, 0.01, 0.1, 1, 10, 50, 100];  ties: ['breslow', 'efron']  (risk) threshold: [0.1, 0.12, …, 0.89, 0.9] | 1 | num_features = 20; alpha = 100; ties = 'efron'; threshold = 0.17 | 0.863 | 0.778 |
|  | 2 | num_features = 20; alpha = 100; ties = ' breslow'; threshold = 0.18 | 0.861 | 0.766 |
|  | 3 | num_features = 20; alpha = 100; ties = 'efron'; threshold = 0.17 | 0.858 | 0.769 |
|  | 4 | num_features = 20; alpha = 100; ties = 'efron'; threshold = 0.17 | 0.857 | 0.765 |
|  | 5 | num_features = 20; alpha = 100; ties = ' breslow'; threshold = 0.18 | 0.861 | 0.779 |

**Supplemental Table 7.** Sample sizes and dementia-event counts for the protected feature subgroups used in the exploratory fairness analysis. “Dementia-positive rows” are rows with observed dementia status = 1. “Unique dementia-positive patients” counts distinct patients with at least one dementia-positive held-out row. These counts should be considered when interpreting subgroup estimates and patient-bootstrap confidence intervals.

| **Protected feature** | **Subgroup** | **Rows** | **Row (%)** | **Unique patients** | **Dementia-positive rows** | **Non-dementia rows** | **Unique dementia-positive patients** | **Dementia prevalence (%)** |
| --- | --- | --- | --- | --- | --- | --- | --- | --- |
| Sex | Female | 79,454 | 59.2 | 26817 | 12714 | 66740 | 10282 | 16.0 |
|  | Male | 54,663 | 40.8 | 20200 | 12253 | 42410 | 9642 | 22.4 |
| Race | White | 109,221 | 81.4 | 37601 | 21080 | 88141 | 16724 | 19.3 |
|  | Black or African American | 18,662 | 13.9 | 6639 | 2470 | 16192 | 2032 | 13.2 |
|  | Asian | 3,364 | 2.5 | 1242 | 587 | 2777 | 446 | 17.4 |
|  | American Indian or Alaska Native | 829 | 0.6 | 416 | 170 | 659 | 160 | 20.5 |
|  | Other | 1512 | 1.1 | 804 | 510 | 1002 | 427 | 33.7 |
| Age | > 65 | 104943 | 78.2 | 35914 | 20058 | 84885 | 15636 | 19.1 |
|  | 45-65 | 27873 | 20.8 | 10561 | 4763 | 23110 | 4181 | 17.1 |
|  | 18-44 | 1301 | 1.0 | 542 | 146 | 1155 | 107 | 11.2 |

# **Calibration and additional model evaluation**

The final selected KNN model was further evaluated using post hoc calibration and additional model evaluation analyses based on pooled cross-fitted predictions from the same-visit and fixed-horizon patient-level analyses. At each horizon, we calculated the observed event proportion (prevalence), mean model-estimated risk score, Brier score, AUC, calibration intercept and calibration slope and constructed quantile-based calibration plots. These analyses describe calibration of the raw KNN score; the score is not an externally validated calibrated probability. Illustrative operating characteristics were also calculated at raw KNN score cutoffs of 0.10, 0.20 and 0.50. These cutoffs show sensitivity/specificity and PPV/NPV trade-offs only and are not calibrated probability thresholds or recommended deployment thresholds. For comparison purposes, we also conducted similar analyses on the Cox model.

**Supplemental Figure 2.** Quantile-based calibration plots for the final KNN model for different horizons, including same-visit classification (h=0) and future prediction (h > 0). Points show the observed event proportion and mean model-estimated risk score within each quantile bin. The dashed diagonal line indicates perfect calibration of the raw model score. Points below the diagonal indicate overestimation of observed event risk, whereas points above the diagonal indicate underestimation.

**Supplemental Figure 3.** Calibration plots of KNN and Cox estimates for different fixed horizons. Each panel contains the same eligible patients for both models at the stated exact horizon. Points show observed event proportions within quantile bins of each model output. The dashed diagonal denotes perfect calibration of the raw model scores.

**Supplemental Table 8.** Confusion-matrix counts and operating characteristics for the final KNN model at illustrative raw-score cutoffs of 0.10, 0.20 and 0.50. The 0.50 threshold corresponds to the stored KNN majority-vote classification. The cutoffs are not calibrated probabilities or recommended deployment thresholds.

| **Horizon** | **Threshold** | **TP** | **FP** | **TN** | **FN** | **sensitivity** | **specificity** | **G-mean** | **Accuracy** | **PPV** | **NPV** |
| --- | --- | --- | --- | --- | --- | --- | --- | --- | --- | --- | --- |
| Same visit (HORIZON = 0) | 0.1 | 15,818 | 13,751 | 17,328 | 120 | 0.992 | 0.558 | 0.744 | 0.705 | 0.535 | 0.993 |
|  | 0.2 | 15,705 | 10,468 | 20,611 | 233 | 0.985 | 0.663 | 0.808 | 0.772 | 0.600 | 0.989 |
|  | 0.5 | 15,152 | 5,918 | 25,161 | 786 | 0.951 | 0.810 | 0.877 | 0.857 | 0.719 | 0.970 |
| Future ≤ 24 months | 0.1 | 1,524 | 7,184 | 8,209 | 56 | 0.965 | 0.533 | 0.717 | 0.573 | 0.175 | 0.993 |
|  | 0.2 | 1,482 | 5,082 | 10,311 | 98 | 0.938 | 0.670 | 0.793 | 0.695 | 0.226 | 0.991 |
|  | 0.5 | 1,276 | 2,216 | 13,177 | 304 | 0.808 | 0.856 | 0.831 | 0.852 | 0.365 | 0.977 |
| Future ≤ 48 months | 0.1 | 2,651 | 5,495 | 4,212 | 89 | 0.968 | 0.434 | 0.648 | 0.551 | 0.325 | 0.979 |
|  | 0.2 | 2,568 | 3,949 | 5,758 | 172 | 0.937 | 0.593 | 0.746 | 0.669 | 0.394 | 0.971 |
|  | 0.5 | 2,166 | 1,568 | 8,139 | 574 | 0.791 | 0.838 | 0.814 | 0.828 | 0.580 | 0.934 |
| Future ≤ 72 months | 0.1 | 3,219 | 4,319 | 1,908 | 67 | 0.980 | 0.306 | 0.548 | 0.539 | 0.427 | 0.966 |
|  | 0.2 | 3,127 | 3,226 | 3,001 | 159 | 0.952 | 0.482 | 0.677 | 0.644 | 0.492 | 0.950 |
|  | 0.5 | 2,628 | 1,275 | 4,952 | 658 | 0.800 | 0.795 | 0.797 | 0.797 | 0.673 | 0.883 |
| Future ≤ 96 months | 0.1 | 3,543 | 3,159 | 785 | 45 | 0.987 | 0.199 | 0.443 | 0.575 | 0.529 | 0.946 |
|  | 0.2 | 3,464 | 2,472 | 1,472 | 124 | 0.965 | 0.373 | 0.600 | 0.655 | 0.584 | 0.922 |
|  | 0.5 | 2,955 | 995 | 2,949 | 633 | 0.824 | 0.748 | 0.785 | 0.784 | 0.748 | 0.823 |
| Future ≤ 120 months | 0.1 | 3,761 | 2,123 | 305 | 25 | 0.993 | 0.126 | 0.353 | 0.654 | 0.639 | 0.924 |
|  | 0.2 | 3,706 | 1,763 | 665 | 80 | 0.979 | 0.274 | 0.518 | 0.703 | 0.678 | 0.893 |
|  | 0.5 | 3,155 | 714 | 1,714 | 631 | 0.833 | 0.706 | 0.767 | 0.784 | 0.815 | 0.731 |

**Supplemental Table 9.** Comprehensive aligned patient-level comparison of KNN and Cox at different future horizons. The table reports observed event proportion, mean KNN raw score, mean Cox estimated risk, AUC, Brier score, calibration intercept and calibration slope. 95% paired patient-bootstrap confidence intervals are also reported.

| **Horizon** | **Evaluable patients** | **Preval-ence** | **Model** | **Estimated model score** | **Sensitivity** | **Specificity** | **G-mean** | **Accuracy** | **AUROC** | **Brier score** | **Calibration intercept** | **Calibration slope** |
| --- | --- | --- | --- | --- | --- | --- | --- | --- | --- | --- | --- | --- |
| 24 | 16,973 | 9.3% | **KNN** | 0.239 (0.235, 0.243) | **0.808 (0.789, 0.827)** | 0.856 (0.851, 0.861) | **0.831 (0.822, 0.842)** | 0.852 (0.846, 0.857) | 0.906 (0.899, 0.913) | 0.102 (0.099, 0.105) | -1.774 (-1.840, -1.712) | 0.944 (0.890, 1.005) |
|  |  |  | **Cox** | 0.073 (0.071, 0.075) | 0.679 (0.658, 0.701) | **0.924 (0.920, 0.928)** | 0.792 (0.779, 0.805) | **0.901 (0.897, 0.906)** | **0.910 (0.902, 0.917)** | **0.061 (0.058, 0.064)** | 1.162 (1.027, 1.301) | 1.406 (1.353, 1.465) |
| 48 | 12,447 | 22.0% | **KNN** | 0.327 (0.322, 0.333) | 0.791 (0.776, 0.805) | **0.838 (0.831, 0.846)** | 0.814 (0.805, 0.823) | **0.828 (0.821, 0.834)** | 0.893 (0.886, 0.899) | 0.120 (0.116, 0.123) | -0.923 (-0.977, -0.869) | 0.994 (0.942, 1.051) |
|  |  |  | **Cox** | 0.173 (0.170, 0.177) | **0.847 (0.834, 0.861)** | 0.808 (0.800, 0.817) | **0.827 (0.820, 0.835)** | 0.817 (0.810, 0.824) | **0.906 (0.900, 0.913)** | **0.104 (0.100, 0.107)** | 1.133 (1.015, 1.258) | 1.467 (1.408, 1.537) |
| 72 | 9,513 | 34.5% | **KNN** | 0.419 (0.413, 0.425) | 0.800 (0.786, 0.813) | **0.795 (0.785, 0.805)** | **0.797 (0.789, 0.806)** | **0.797 (0.789, 0.804)** | 0.877 (0.870, 0.885) | 0.140 (0.136, 0.144) | -0.514 (-0.571, -0.461) | 1.040 (0.977, 1.102) |
|  |  |  | **Cox** | 0.264 (0.259, 0.269) | **0.906 (0.897, 0.916)** | 0.679 (0.667, 0.690) | 0.784 (0.776, 0.792) | 0.757 (0.748, 0.766) | **0.903 (0.896, 0.910)** | **0.132 (0.128, 0.137)** | 1.218 (1.097, 1.346) | 1.476 (1.406, 1.553) |
| 96 | 7,532 | 47.6% | **KNN** | 0.497 (0.491, 0.503) | 0.824 (0.812, 0.836) | **0.748 (0.734, 0.761)** | **0.785 (0.776, 0.793)** | **0.784 (0.775, 0.793)** | 0.863 (0.855, 0.871) | **0.152 (0.148, 0.156)** | -0.145 (-0.205, -0.087) | 1.128 (1.057, 1.200) |
|  |  |  | **Cox** | 0.354 (0.347, 0.360) | **0.937 (0.928, 0.945)** | 0.571 (0.555, 0.586) | 0.731 (0.721, 0.742) | 0.745 (0.735, 0.755) | **0.902 (0.895, 0.908)** | 0.153 (0.148, 0.158) | 1.396 (1.277, 1.525) | 1.495 (1.419, 1.586) |
| 120 | 6,214 | 60.9% | **KNN** | 0.556 (0.549, 0.562) | 0.833 (0.822, 0.845) | **0.706 (0.687, 0.725)** | **0.767 (0.756, 0.779)** | **0.784 (0.773, 0.794)** | 0.851 (0.842, 0.861) | **0.156 (0.151, 0.160)** | 0.270 (0.206, 0.327) | 1.280 (1.207, 1.363) |
|  |  |  | **Cox** | 0.450 (0.442, 0.458) | **0.966 (0.960, 0.971)** | 0.449 (0.428, 0.468) | 0.658 (0.643, 0.672) | 0.764 (0.752, 0.773) | **0.903 (0.896, 0.910)** | 0.164 (0.158, 0.169) | 1.550 (1.432, 1.685) | 1.491 (1.397, 1.591) |

# **Sensitivity Analyses**

These sensitivity analyses were conducted to assess whether the main findings were robust to key modelling and evaluation choices in the dataset. Specifically, we examined whether the use of SMOTE-NC within HORIZON groups materially changed the performance of the final KNN model, presented secondary-analysis performance estimates based on patient-visit row level, including whether these were materially affected by patients contributing multiple observed horizon rows.

**Supplemental Table 10.** Inverse probability of censoring weighted sensitivity analysis of fixed-horizon KNN discrimination and prediction error. Fold-specific Kaplan-Meier censoring distributions were estimated from outer-training patients and applied to the corresponding held-out patients. Time-dependent AUC and IPCW Brier score are shown with censoring-survival, weight and effective sample size diagnostics, before 99th-percentile weight truncation. The results produced after 99th-percentile weight truncation are not shown as they are exactly the same.

| Horizon | Patients | Cases by horizon | Dynamic controls | Censored before horizon | IPCW AUC | IPCW Brier score | Minimum fold specific G(T) | Maximum raw weight | Raw weight p99 | Maximum analysis weight | Effective sample size |
| --- | --- | --- | --- | --- | --- | --- | --- | --- | --- | --- | --- |
| 24 | 31079 | 1580 | 15669 | 13830 | 0.891 | 0.116 | 0.547 | 1.827 | 1.827 | 1.827 | 17217.1 |
| 48 | 31079 | 2740 | 9844 | 18495 | 0.878 | 0.125 | 0.378 | 2.648 | 2.648 | 2.648 | 12298.4 |
| 72 | 31079 | 3286 | 6224 | 21569 | 0.855 | 0.147 | 0.256 | 3.913 | 3.913 | 3.913 | 8755.4 |
| 96 | 31079 | 3588 | 3949 | 23542 | 0.833 | 0.166 | 0.173 | 5.792 | 5.792 | 5.792 | 6229.9 |
| 120 | 31079 | 3786 | 2421 | 24872 | 0.819 | 0.178 | 0.112 | 8.907 | 8.907 | 8.907 | 4322.3 |

**Supplemental Table 11.** No-SMOTE-NC sensitivity analysis for the final KNN model. To assess the effect of horizon-wise synthetic oversampling, we repeated the final KNN model evaluation with SMOTE-NC disabled while retaining the same grouped held-out evaluation framework (KNN was again tuned using the same set of 120 hyperparameters). Values are point estimates (95% patient-bootstrap confidence intervals; 1,000 replicates).

|  | **G-mean** | | **Sensitivity** | | **Specificity** | |
| --- | --- | --- | --- | --- | --- | --- |
| **Horizon** | **No SMOTENC** | **SMOTENC** | **No SMOTENC** | **SMOTENC** | **No SMOTENC** | **SMOTENC** |
| 0 | 0.877 (0.874, 0.880) | 0.877 (0.874, 0.880) | 0.824 (0.818, 0.830) | 0.951 (0.947, 0.954) | 0.933 (0.930, 0.935) | 0.810 (0.805, 0.814) |
| 24 | 0.607 (0.586, 0.626) | 0.831 (0.822, 0.842) | 0.379 (0.354, 0.404) | 0.808 (0.789, 0.827) | 0.971 (0.968, 0.974) | 0.856 (0.850, 0.861) |
| 48 | 0.589 (0.575, 0.603) | 0.814 (0.806, 0.822) | 0.357 (0.339, 0.374) | 0.791 (0.775, 0.805) | 0.973 (0.970, 0.977) | 0.838 (0.831, 0.845) |
| 72 | 0.576 (0.562, 0.590) | 0.797 (0.789, 0.806) | 0.341 (0.325, 0.357) | 0.800 (0.786, 0.813) | 0.973 (0.968, 0.977) | 0.795 (0.785, 0.806) |
| 96 | 0.560 (0.546, 0.573) | 0.785 (0.776, 0.794) | 0.321 (0.306, 0.337) | 0.824 (0.811, 0.836) | 0.976 (0.971, 0.980) | 0.748 (0.734, 0.762) |
| 120 | 0.541 (0.529, 0.555) | 0.767 (0.756, 0.778) | 0.299 (0.285, 0.314) | 0.833 (0.822, 0.845) | 0.980 (0.974, 0.985) | 0.706 (0.689, 0.724) |

**Supplemental Table 12.** Secondary cumulative observed-row performance of the final KNN model. For target horizons greater than zero, the estimates pool all observed held-out rows satisfying 0 < HORIZON ≤ the target horizon.

|  | **Horizon** | | **Sensitivity** | | **Specificity** | | **ROC AUC** | | **Accuracy** | | **Test sample size (median)** | |
| --- | --- | --- | --- | --- | --- | --- | --- | --- | --- | --- | --- | --- |
| Same-visit diagnosis | 0 | | 0.949 (0.005) | | 0.813 (0.009) | | 0.959 (0.002) | | 0.859 (0.005) | | 9,422 | |
| Incident-dementia prediction | 24 | | 0.791 (0.032) | | 0.835 (0.007) | | 0.887 (0.010) | | 0.832 (0.005) | | 4,757 | |
|  | 48 | | 0.771 (0.037) | | 0.838 (0.006) | | 0.881 (0.009) | | 0.832 (0.002) | | 9,664 | |
|  | 72 | | 0.753 (0.033) | | 0.830 (0.007) | | 0.871 (0.008) | | 0.822 (0.004) | | 12,883 | |
|  | 96 | | 0.743 (0.030) | | 0.822 (0.008) | | 0.862 (0.008) | | 0.814 (0.004) | | 14,889 | |
|  | 120 | | 0.736 (0.030) | | 0.814 (0.007) | | 0.855 (0.008) | | 0.806 (0.004) | | 16,136 | |
|  | Overall (H>0) | | 0.723 (0.025) | | 0.808 (0.007) | | 0.847 (0.007) | | 0.799 (0.004) | | 17,402 | |
| Overall | | 0.867 (0.012) | | 0.809 (0.007) | | 0.921 (0.002) | | 0.820 (0.004) | | 26,824 | |  |

**Supplemental Table 13.** Secondary cumulative observed-row sensitivity analysis comparing conventional row weighting with equal total weighting per patient.

|  |  | **G-mean** | | | **Sensitivity** | | | **Specificity** | | | **ROC AUC** | | | **Accuracy** | | |
| --- | --- | --- | --- | --- | --- | --- | --- | --- | --- | --- | --- | --- | --- | --- | --- | --- |
|  | **Horizon** | **Row-level** | **Patient-weighted** | **Diff** | **Row-level** | **Patient-weighted** | **Diff** | **Row-level** | **Patient-weighted** | **Diff** | **Row-level** | **Patient-weighted** | **Diff** | **Row-level** | **Patient-weighted** | **Diff** |
| **Same-visit diagnosis** | 0 | 0.878 | 0.878 | <  0.000 | 0.949 | 0.949 | <  0.000 | 0.813 | 0.813 | <  0.000 | 0.959 | 0.959 | <  0.000 | 0.859 | 0.859 | < 0.000 |
| **Future risk prediction** | 24 | 0.813 | 0.810 | -0.002 | 0.791 | 0.787 | -0.004 | 0.835 | 0.834 | 0.000 | 0.887 | 0.886 | -0.001 | 0.832 | 0.831 | -0.001 |
|  | 48 | 0.804 | 0.798 | -0.006 | 0.771 | 0.764 | -0.007 | 0.838 | 0.834 | -0.004 | 0.881 | 0.878 | -0.004 | 0.832 | 0.827 | -0.004 |
|  | 72 | 0.790 | 0.788 | -0.002 | 0.753 | 0.755 | 0.002 | 0.830 | 0.824 | -0.006 | 0.871 | 0.869 | -0.002 | 0.822 | 0.816 | -0.006 |
|  | 96 | 0.781 | 0.783 | 0.002 | 0.743 | 0.752 | 0.009 | 0.822 | 0.816 | -0.006 | 0.862 | 0.864 | 0.001 | 0.814 | 0.809 | -0.005 |
|  | 120 | 0.774 | 0.779 | 0.006 | 0.736 | 0.749 | 0.013 | 0.814 | 0.811 | -0.003 | 0.855 | 0.860 | 0.005 | 0.806 | 0.804 | -0.002 |
|  | Overall H>0 | 0.764 | 0.775 | 0.011 | 0.723 | 0.745 | 0.022 | 0.808 | 0.807 | <  0.000 | 0.847 | 0.856 | 0.009 | 0.799 | 0.800 | 0.001 |

# **Illustrative guide to the Streamlit application**

The screenshots below illustrate the main workflow of the Streamlit application, from the landing page and data entry form to the prediction output, SHAP explanation, and the summary of final inputs used by the model. Baseline values are provided for all inputs, but the model can also handle missing values (median for continuous and ordinal variables, and the mode for nominal variables).

We also measured application prototype runtime and storage requirements. The full repository package is approximately 54 MB, and the compressed model artefact is approximately 44 MB. On the local Windows 11 laptop described in Section 2.7, loading the KNN model pipeline took 0.94 seconds, loading the SHAP explainer took 0.21 seconds, generating the model classification and risk score for one case took 0.86 seconds, and generating the SHAP explanation took 40.75 seconds. On the AWS EC2 t2.micro instance used for the demonstration application, a prediction without SHAP was generated in under 10 seconds and a prediction with SHAP in under two minutes.

|  | | **Supplemental Figure 4.** Illustrative guide to the Streamlit application input workflow. The four panels show the landing page (panel A), the start of numerical data entry (B), independence and motor assessment inputs (C), and functional activity questions (D) before submission. | |
| --- | --- | --- | --- |
|  | **Supplemental Figure 5.** Illustrative guide to the application output pages. The upper panel shows the prediction summary (A), and the lower panels show the SHAP-based local explanation (B) and the summary table of supplied inputs and final values used by the model, including imputed values where applicable (C). | |  |

**Supplemental Table 14.** Comparison of proposed flexible-horizon machine learning model with related works across different time horizons.

| **Horizon** | **Research work** | **Number of features** | **Sensitivity** | **Specificity** | **G-mean^1^** | **AUC** |
| --- | --- | --- | --- | --- | --- | --- |
| 0 (diagnosis) | Qiu et al. [1] ^2^ | 65 | 0.53 | **0.94** | 0.71 | - |
|  | **This study** | 20 | **0.95** | 0.81 | **0.88** | 0.96 |
| 12 | Bucholc et al. [2] ^3^ | 16 | **0.93** | 0.58 | 0.73 | 0.76 |
|  | **This study** | 20 | 0.82 | **0.84** | **0.83** | **0.90** |
| 29 | James et al. [3] | 22 | 0.45 | **0.97** | 0.66 | **0.92** |
|  | **This study** | 20 | **0.80** | 0.86 | **0.83** | 0.90 |
| 144 | Twait et al. [4] ^4^ | 16 | 0.61 | 0.69 | 0.65 | 0.72 |
|  | **This study** | 20 | **0.81** | **0.71** | **0.76** | **0.84** |

^1^ Since the G-mean (geometric mean of sensitivity and specificity) was not reported in these studies, we calculated it using the reported sensitivity and specificity for comparison; ^2^ We report their CatBoost model for dementia classification based on non-imaging variables; ^3^ The Bucholc et al.’s model predicts conversion from mild cognitive impairment to dementia; ^4^ This is the only study reported here that trained its model on a dataset other than NACC UDS.

# **References**

[1] Qiu S, Miller MI, Joshi PS, Lee JC, Xue C, Ni Y, et al. Multimodal deep learning for Alzheimer’s disease dementia assessment. Nature Communications. 2022;13:3404. doi:<https://doi.org/10.1038/s41467-022-31037-5>.

[2] Bucholc M, Titarenko S, Ding X, Canavan C, Chen T. A hybrid machine learning approach for prediction of conversion from mild cognitive impairment to dementia. Expert Systems with Applications. 2023;217:119541. doi:<https://doi.org/10.1016/j.eswa.2023.119541>.

[3] James C, Ranson JM, Everson R, Llewellyn DJ. Performance of Machine Learning Algorithms for Predicting Progression to Dementia in Memory Clinic Patients. JAMA Network Open. 2021;4:e2136553-e. doi:<https://doi.org/10.1001/jamanetworkopen.2021.36553>.

[4] Twait EL, Andaur Navarro CL, Gudnason V, Hu Y-H, Launer LJ, Geerlings MI. Dementia prediction in the general population using clinically accessible variables: a proof-of-concept study using machine learning. The AGES-Reykjavik study. BMC Medical Informatics and Decision Making. 2023;23:168. doi:<https://doi.org/10.1186/s12911-023-02244-x>.
